# Efficient C–N coupling for urea electrosynthesis on defective Co_3_O_4_ with dual-functional sites†

**DOI:** 10.1039/d3sc06579k

**Published:** 2024-01-18

**Authors:** Pengsong Li, Qinggong Zhu, Jiyuan Liu, Tianbin Wu, Xinning Song, Qinglei Meng, Xinchen Kang, Xiaofu Sun, Buxing Han

**Affiliations:** a Beijing National Laboratory for Molecular Sciences, CAS Laboratory of Colloid and Interface and Thermodynamics, CAS Research/Education Center for Excellence in Molecular Sciences, Center for Carbon Neutral Chemistry, Institute of Chemistry, Chinese Academy of Sciences Beijing 100190 China qgzhu@iccas.ac.cn hanbx@iccas.ac.cn; b School of Chemistry and Chemical Engineering, University of Chinese Academy of Sciences Beijing 100049 China; c Shanghai Key Laboratory of Green Chemistry and Chemical Processes, School of Chemistry and Molecular Engineering, East China Normal University Shanghai 200062 China

## Abstract

Urea electrosynthesis under ambient conditions is emerging as a promising alternative to conventional synthetic protocols. However, the weak binding of reactants/intermediates on the catalyst surface induces multiple competing pathways, hindering efficient urea production. Herein, we report the synthesis of defective Co_3_O_4_ catalysts that integrate dual-functional sites for urea production from CO_2_ and nitrite. Regulating the reactant adsorption capacity on defective Co_3_O_4_ catalysts can efficiently control the competing reaction pathways. The urea yield rate of 3361 mg h^−1^ g_cat_^−1^ was achieved with a corresponding faradaic efficiency (FE) of 26.3% and 100% carbon selectivity at a potential of −0.7 V *vs.* the reversible hydrogen electrode. Both experimental and theoretical investigations reveal that the introduction of oxygen vacancies efficiently triggers the formation of well-matched adsorption/activation sites, optimizing the adsorption of reactants/intermediates while decreasing the C–N coupling reaction energy. This work offers new insights into the development of dual-functional catalysts based on non-noble transition metal oxides with oxygen vacancies, enabling the efficient electrosynthesis of essential C–N fine chemicals.

## Abstract 2

Defective Co_3_O_4_ catalysts featuring dual-functional sites were synthesized using a facile thermal annealing strategy to enhance the adsorption of reactants/intermediates and facilitate effective C–N coupling for urea production from CO_2_ and nitrite.

## Graphical abstract

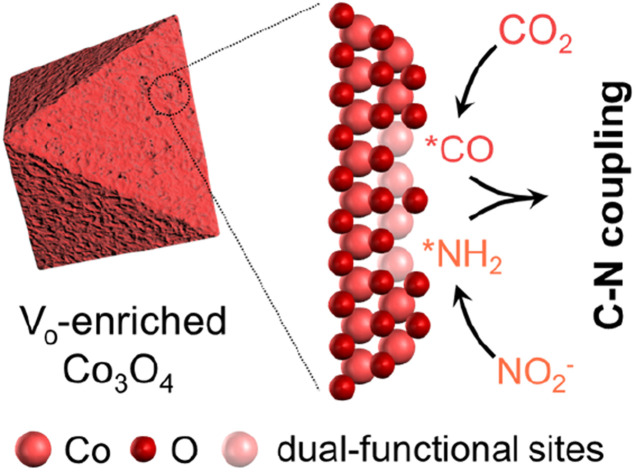

## Introduction

Urea is widely used as a fertilizer, as well as a chemical raw material for medicines and pesticides. However, traditional industrial urea synthesis, relying on the reaction of CO_2_ and NH_3_, involves harsh conditions and leaves a significant carbon footprint,^1,2^ necessitating the pursuit of greener, more energy-efficient, and cost-effective alternatives.^3,4^ Electrochemical urea synthesis from N-integrated electrocatalytic CO_2_ reduction is gradually emerging as a promising alternative to reform the conventional urea industry.^5–7^ Most strikingly, emerging electrocatalytic co-reduction of N_2_ and CO_2_ in an aqueous solution has been demonstrated as a feasible method for urea synthesis.^8–12^ However, the inherent chemical inertness of N_2_, such as high triple bond dissociation energy (941 kJ mol^−1^) and low solubility (∼0.02 v/v, 1 atm, 25 °C), poses a considerable limitation on urea yield rates.

Alternatively, N-integrated electrochemical CO_2_ reduction can be performed with nitrate/nitrite, which is a widely existing contaminant in the wastewater and poses severe risks to both public health and the environment. In particular, using nitrite as a N-containing reactant for urea electrosynthesis is highly desirable due to its merits of superior solubility (*e.g.* KNO_2_, 2.81 g mL^−1^, 0 °C) and lower N=O bond dissociation energy (204 kJ mol^−1^), which could potentially result in higher performance for urea synthesis.^13^ Some pioneer works have shown that urea can be synthesized from electrochemically coupling CO_2_ and nitrate/nitrite.^14–20^ For example, the In(OH)_3_–S catalyst could promote co-electrolysis of CO_2_ and nitrate to urea with an average yield rate of 533.1 mg h^−1^ g_cat_^−1^ and a Faradaic efficiency (FE) of 53.4% at the potential of −0.6 V *vs.* the reversible hydrogen electrode (RHE).^21^ Over a Cu_1_–CeO_2_ catalyst, the yield rate of urea could reach 52.84 mmol h^−1^ g_cat_^−1^ at −1.6 V *vs.* RHE.^22^ Low-valence Cu^*δ*+^-doped anatase TiO_2_ was employed as an electrocatalyst for the co-electrolysis of CO_2_ and nitrite ions to urea with a yield rate of 20.8 μmol h^−1^ at a low potential of −0.4 V *vs.* RHE.^23^ In another study, a Te-doped Pd nanocrystal was utilized to enhance the electrochemical urea production by coupling CO_2_ and nitrite reduction with nearly 12.2% FE and 88.7% N atom efficiency.^24^ Despite substantial progress having been made in urea synthesis, there still remains a significant challenge in improving urea FE and yield rate. This is because the parallel CO_2_ reduction reaction, nitrate/nitrite reduction reaction and inescapable hydrogen evolution reaction (HER) strongly compete with the desirable C–N coupling reaction, leading to low selectivity of the urea product. The primary obstacle lies in the suppression of selective hydrogenation of intermediate species to unwanted by-products and the hindrance of the unfavorable HER to facilitate C–N coupling for urea production.

To address this, we proposed a two-step thermal annealing strategy to synthesize defective Co_3_O_4_ catalysts, integrating dual-functional sites to favor urea electrosynthesis. We discovered that the presence of oxygen vacancies (V_o_) could construct well-matched adsorption/activation sites, which strengthened the adsorption of CO_2_ and NO_2_^−^ reactants. The modulated local environment on Co atoms near the oxygen vacancies, serving as the dual-functional sites, not only strengthened the adsorption of intermediates but also decreased the C–N coupling resistance in urea synthesis. Notably, when using defective Co_3_O_4_ obtained under suitable treatment conditions as an electrocatalyst, an impressive urea yield rate of 3361 mg h^−1^ g_cat_^−1^ was achieved with a corresponding FE of 26.3% and 100% carbon selectivity at the potential of −0.7 V *vs.* RHE.

## Results and discussion

**Fig. 1:**
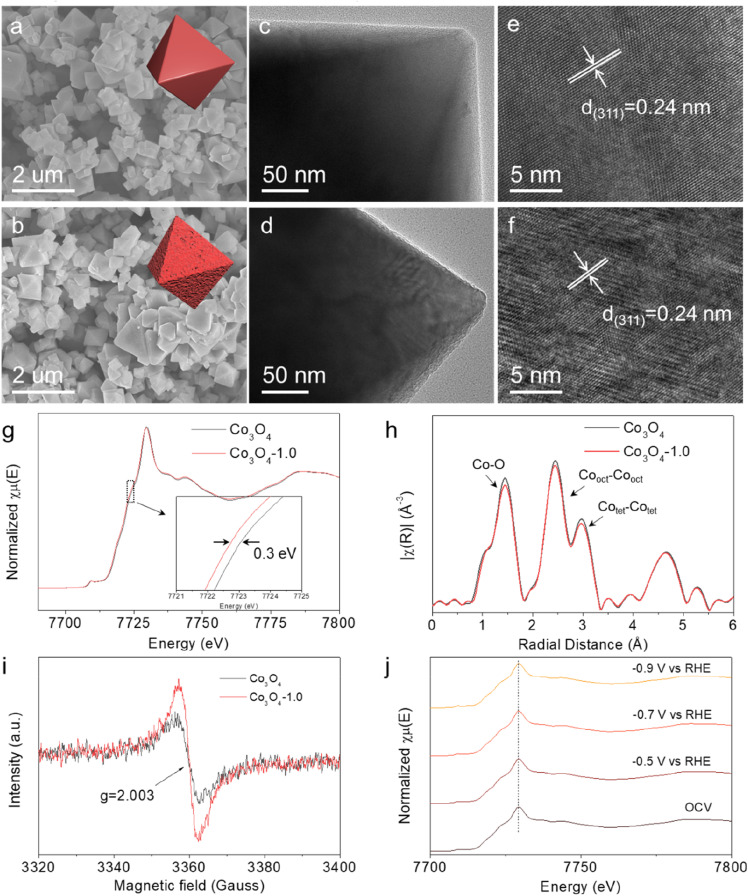
SEM images of (a) pristine Co_3_O_4_ and (b) Co_3_O_4_-1.0. TEM images of (c) pristine Co_3_O_4_ and (d) Co_3_O_4_-1.0. HRTEM images of (e) pristine Co_3_O_4_ and (f) Co_3_O_4_-1.0. (g) XANES and (h) Fourier-transformed EXAFS spectra of the Co K-edge over pristine Co_3_O_4_ and Co_3_O_4_-1.0. (i) EPR spectra of pristine Co_3_O_4_ and Co_3_O_4_-1.0. (j) Operando XANES under the electrochemical conditions of Co K-edge over Co_3_O_4_-1.0. OCV stands for open-circuit voltage.

We used a two-step thermal annealing strategy to synthesize defective Co_3_O_4_ catalysts with tunable V_o_ concentrations. Briefly, Co(NO_3_)_2_·6H_2_O and NaCl were first mixed in an agate mortar, followed by adding NaOH solution for sufficient grinding and solvent removal. The resulting solid solution was then directly pyrolysed under an Ar atmosphere to get the pristine Co_3_O_4_, in which the V_o_ concentrations could be tuned by thermal treatment under a reducing atmosphere (H_2_/Ar) for different times (0.5, 1.0 and 1.5 h). The as-synthesized catalysts were denoted as Co_3_O_4_-0.5, Co_3_O_4_-1.0, and Co_3_O_4_-1.5, respectively. We commenced our characterization using scanning electron microscopy (SEM). As shown in Fig. 1a and b and S1,† both the pristine and reduced thermally treated Co_3_O_4_ had nanosized and octahedral structures, indicating that the reducing thermal annealing process did not affect the initial morphology. The morphological and structural information was further studied by transmission electron microscopy (TEM) and high-resolution transmission electron microscopy (HRTEM). Typically, the TEM images in Fig. 1c and d demonstrated that Co_3_O_4_-1.0 had a rougher surface than pristine Co_3_O_4_, which could be attributed to the formation of high V_o_ concentration on the catalyst surface. The HRTEM images in Fig. 1e and f showed that the lattice fringe of the Co_3_O_4_ nanoparticle was 0.24 nm, which is ascribed to the lattice plane distance of the (311) plane of Co_3_O_4_. The main difference was that the pristine Co_3_O_4_ featured a flatter crystal surface (Fig. 1e) than Co_3_O_4_-1.0 (Fig. 1f). The normalized Co K-edge X-ray absorption near edge structure (XANES) spectra (Fig. 1g) revealed a slightly reduced average valence state of the Co species in Co_3_O_4_-1.0, indicating that V_o_ can manipulate the electronic state of the nearby Co atoms. Additionally, the Co K-edge extended X-ray absorption fine structure (EXAFS) spectra of pristine Co_3_O_4_ and Co_3_O_4_-1.0 demonstrated three distinct peaks (Fig. 1h), which were identified as Co–O, tetrahedral Co–Co, and octahedral Co–Co bonds, respectively. Comparably, the intensity of the Co–O bond in Co_3_O_4_-1.0 was lower than that in the initial Co_3_O_4_, suggesting a reduced oxygen coordination number for the Co atoms in Co_3_O_4_-1.0. This reduction is attributed to the introduction of additional V_o_ in Co_3_O_4_-1.0.^25,26^

The X-ray diffraction (XRD) patterns of the catalysts are shown in Fig. S2.† In detail, the peaks located at 31, 37, 44, 59 and 65° can be indexed to the (220), (311), (400), (511) and (440) crystalline planes of Co_3_O_4_ (JCPDS: PDF#43-1003), respectively, confirming the successful synthesis of Co_3_O_4_. The electron paramagnetic resonance (EPR) spectra were recorded to identify V_o_. The electron deficient oxygen species exhibited an EPR signal at *g* = 2.003 (Fig. 1i and S3†).^27,28^ The intensity increased with increasing annealing time, revealing a higher V_o_ concentration in the Co_3_O_4_-1.0 catalyst than in the pristine Co_3_O_4_ catalyst. Furthermore, X-ray photoelectron spectroscopy (XPS) measurement analysis was performed to investigate the chemical states and compositions of the catalysts. The XPS spectra of O 1s (Fig. S4†) verified that the lattice oxygen (O_1_) and oxygen vacancies (O_2_) existed in all catalysts.^29,30^ The O_2_/O_1_ ratio means the ratio of O_2_ and O_1_ area in the O 1s spectra, and followed the sequence of pristine Co_3_O_4_ < Co_3_O_4_-0.5 < Co_3_O_4_-1.0 < Co_3_O_4_-1.5, suggesting the presence of more V_o_ species in the Co_3_O_4_-1.5 catalyst, which was consistent with the EPR results. Fig. S5† displayed the impact of V_o_ on the electronic structure of Co_3_O_4_. The peaks at around 780 eV (Co 2p_3/2_) and 795 eV (Co 2p_1/2_) retained the characteristic feature of Co species.^25^ Apparently, the Co^2+^/Co^3+^ ratio increased from 0.85 to 1.54 with increasing V_o_ concentration, suggesting a decrease in Co valence state on the Co_3_O_4_ surface after thermal treatment under the reducing atmosphere. Interestingly, we found a positive linear correlation between the ratio of Co^2+^/Co^3+^ and the ratio of O_2_/O_1_ (Fig. S6†), indicating that the V_o_ level in the catalysts was directly correlated with the electron cloud density of Co atoms, which is critical for the electrocatalytic activity.

The structural stability of electrocatalyst is a crucial indicator when assessing its performance. Therefore, we first carried out operando X-ray absorption spectroscopy (XAS) to investigate the structural and valence state changes of Co element under the electrochemical reaction conditions. In the operando XAS measurement, the potential was decreased from open-circuit voltage (OCV) to −0.5 V, −0.7 V, and −0.9 V *vs.* RHE. Fig. 1j and S7† demonstrate that there was no energy shift on the Co K-edge in the XANES spectra as the applied potential decreased. Similarly, no changes were observed in the coordination environment of Co atoms in the EXAFS spectra (Fig. S8†). These results collectively suggest that the local structure and electronic state of Co species in Co_3_O_4_-1.0 remained stable during the electrochemical reaction process.

**Fig. 2:**
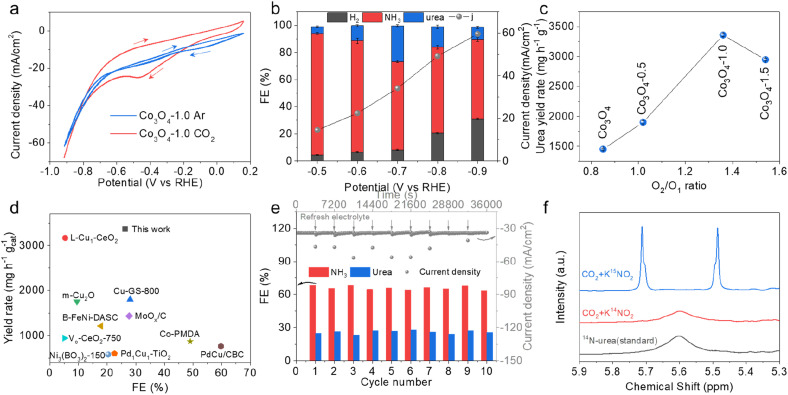
(a) CV curves of Co_3_O_4_-1.0 at the scan rate of 50 mV s^−1^ in Ar or CO_2_ saturated electrolyte. (b) The FE of major products and total current densities over the Co_3_O_4_-1.0 electrocatalyst in CO_2_ saturated 0.2 M KHCO_3_ + 0.02 M KNO_2_ electrolyte. (c) The urea yield rate *vs.* the ratio of O_2_/O_1_ at the potential of −0.7 V (*vs.* RHE). (d) Comparison of the FE (%) of urea and yield rate (mg h^−1^ g_cat_^−1^) of urea of Co_3_O_4_-1.0 with those of state-of-the-art catalysts for application in urea synthesis. (e) Stability tests of Co_3_O_4_-1.0 at the potential of −0.7 V (*vs.* RHE). (f) ^1^H NMR spectra obtained by using K^14^NO_2_ and K^15^NO_2_ as the reactants with CO_2_ and a standard ^14^N-urea sample.

The electrocatalytic activity of the as-prepared catalysts for application in urea synthesis was evaluated in an H-type cell. Cyclic voltammetry (CV) was carried out to preliminarily assess the performance of the Co_3_O_4_-1.0 in Ar or CO_2_ saturated 0.2 M KHCO_3_ + 0.02 M KNO_2_ electrolyte. As shown in Fig. 2a, in the presence of CO_2_, Co_3_O_4_-1.0 exhibited larger current density responses during the forward scan from −0.2 V to −0.8 V *vs.* RHE, resulting from simultaneous electroreduction of CO_2_ and NO_2_^−^. Subsequently, chronoamperometry tests were conducted at applied potentials ranging from −0.5 to −0.9 V *vs.* RHE to analyze the FE and the yield of products. The yield of urea was quantified using the well-established urease decomposition and indophenol blue spectrophotometric method (Fig. S9 and S10†).^15,25,31^ The performances of urea electrosynthesis at different applied potentials are displayed in Fig. 2b and S11.† It can be found that Co_3_O_4_-1.0 mainly yielded H_2_, NH_3_, and urea with a combined FE of around 100%. With increasing applied potential, the current density and the FE of urea are increased. At the potential of −0.7 V *vs.* RHE, the FE of urea could reach 26.3% with the current density of 34.1 mA cm^−2^ over the Co_3_O_4_-1.0 catalyst. When the potential was beyond −0.7 V *vs.* RHE, the FE of urea decreased, mainly due to the highly competitive HER. It is noteworthy that no other reduction products from CO_2_ were detected across the potential range, indicating that the catalyst demonstrated a 100% carbon selectivity for C–N coupling. Fig. S12–S14† show the FE of urea and current density of comparative catalysts (pristine Co_3_O_4_, Co_3_O_4_-0.5, and Co_3_O_4_-1.5) within the potential range from −0.5 V to −0.9 V *vs.* RHE. This indicates that the product distributions of NH_3_, urea, and H_2_ were correlated with the V_o_ concentration in the catalysts, in which Co_3_O_4_-1.0 with an appropriate V_o_ concentration exhibited the best performance for urea formation among all the catalysts. At the optimized condition, the urea yield rate over the Co_3_O_4_-1.0 catalyst could reach 3361 mg h^−1^ g_cat_^−1^ (Fig. 2c), which was nearly 2.3 times that of pristine Co_3_O_4_. For the pristine Co_3_O_4_ catalyst, the FE of urea was only 13.7% with a limited urea yield rate of 1450 mg h^−1^ g_cat_^−1^. Comparison with the state-of-the-art catalysts for urea synthesis is presented in Fig. 2d and Table S1.† It shows that Co_3_O_4_-1.0 was highly efficient as an electrocatalyst for C–N coupling to produce urea and it could reach high FE and yield rate at a low reduction potential.

Remarkably, in the ten consecutive cycles at −0.7 V *vs.* RHE, the Co_3_O_4_-1.0 achieved long-term stability with no obvious decays in current density, and the FE of urea and NH_3_ could be held around 25% and 65%, respectively, during the whole electrolysis process (Fig. 2e). Moreover, the XRD, XPS, and TEM measurements showed that the crystal structure, valence states, V_o_, and morphology were well preserved after the long-term electrolysis (Fig. S15–S17†), indicating the excellent electrochemical and structural stability of the Co_3_O_4_-1.0 catalyst.

To identify the source of urea production, we performed the isotope-labelling experiments in CO_2_-saturated 0.2 M KHCO_3_ + 0.02 M K^15^NO_2_ electrolytes. Fig. 2f illustrates the ^1^H NMR spectrum of the electrolyte with K^15^NO_2_, displaying the typical double peaks of ^15^N-urea at 5.48 and 5.71 ppm, while the ^1^H NMR spectrum of the electrolyte with K^14^NO_2_ only exhibits a single peak of ^14^N-urea at 5.60 ppm. The ^1^H NMR results confirm that there is urea production in the coupling electrolysis of CO_2_ and ^14^NO_2_^−^ on Co_3_O_4_-1.0. Furthermore, we employed the ^1^H NMR technique (Fig. S18 and S19†) to quantitatively measure the generated urea and concurrently validate the accuracy of the UV-vis method. The obtained results (Fig. S20†) demonstrate a consistent urea concentration measurement between the ^1^H NMR and UV-vis methods, confirming the accuracy of the employed quantitative method.

Considering the experimental observations above, we think that the high electrocatalytic activity of the Co_3_O_4_-1.0 catalyst could be attributed to the generation of abundant active sites in defective Co_3_O_4_, which was kinetically favorable for the reaction. To verify the hypothesis, we estimated the electrochemical active surface area (ECSA) of various catalysts through electrochemical double-layer capacitance (*C*_dl_) measurements. Fig. S21† shows the cyclic voltammetry (CV) curves with various scan rates at the non-faradaic region for the catalysts. The linear slopes in Fig. S22† show that the Co_3_O_4_-1.0 had a larger ECSA, suggesting that the higher V_o_ concentration was responsible for the generation of more active sites. After normalizing the urea yield rate to ECSA (Fig. S23†), Co_3_O_4_-1.0 still exhibited the largest urea yield rate (1832 mg h^−1^ g_cat_^−1^) at the potential of −0.7 V *vs.* RHE, which indicates that the V_o_ concentration regulation could also improve the intrinsic activity of urea production over the catalysts. In addition, the electrochemical impedance spectrum (EIS, Fig. S24†) was recorded to probe the effect of defects on the charge transport kinetics. It showed that charge resistance (*R*_ct_) on Co_3_O_4_-1.0 was much lower than that on pristine Co_3_O_4_, which is favorable for enhancing the reaction rate.

**Fig. 3:**
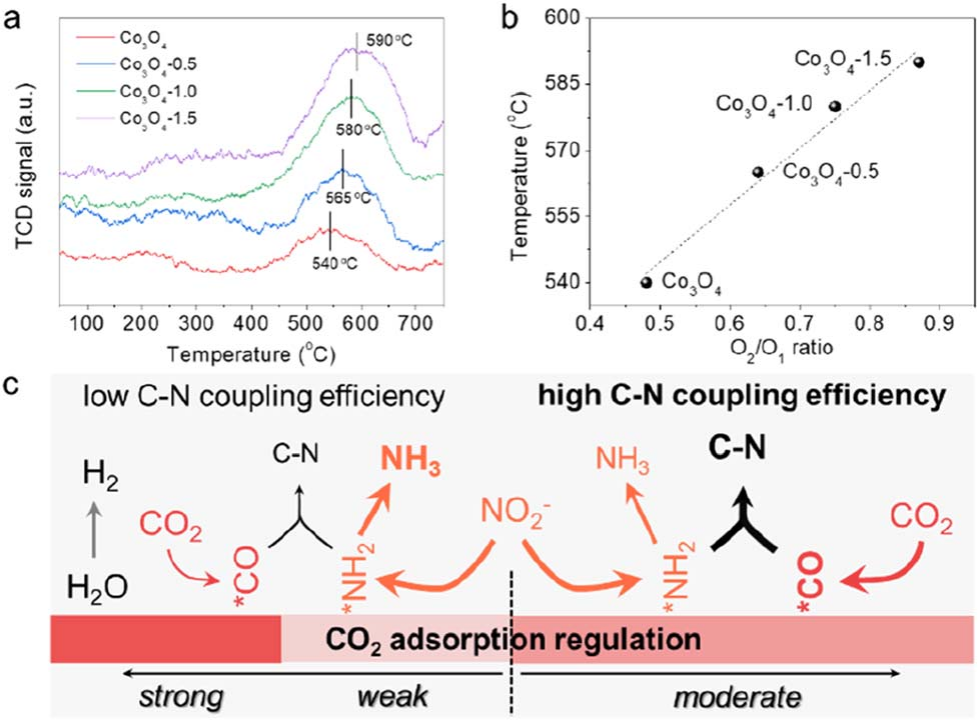
(a) CO_2_-TPD plots for different catalysts normalized to ECSA. (b) The temperature of CO_2_ desorption peak *vs.* the ratio of O_2_/O_1_ over different catalysts. (c) Schematic diagram of CO_2_ adsorption regulation for efficient C–N coupling.

The defect-engineering would serve as a promising strategy for simultaneously enhancing the chemisorption capability of these inert molecules and regulating the electronic structure of the catalyst to construct dual-functional sites. As effective chemisorption of reactants is essential for the initial stage of urea electrosynthesis, we turn to screen the effect of defects on the CO_2_ chemical adsorption properties. The improved adsorption capacity of CO_2_ molecules on V_o_-enriched Co_3_O_4_ can be verified *via* the CO_2_ temperature programmed desorption (CO_2_-TPD) spectra (Fig. 3a) normalized to ECSA. Comparably, all the defective Co_3_O_4_ catalysts exhibited stronger binding strength and larger adsorption peaks than the pristine Co_3_O_4_. Typically, it was exhibited that CO_2_ desorption occurred at around 580 °C on the Co_3_O_4_-1.0 catalyst, indicating that it had a strong CO_2_ adsorption site with excellent CO_2_ adsorption ability. In contrast, the CO_2_ desorption peak of the pristine Co_3_O_4_ catalyst was at around 540 °C, which was significantly weaker than that of the Co_3_O_4_-1.0 catalyst. We then correlated the temperature of the CO_2_ desorption peak with the concentration of V_o_ (the ratio of O_2_/O_1_) to investigate the correlation between V_o_ and CO_2_ adsorption capacity (Fig. 3b). This indicates that an increase in the concentration of V_o_ in the catalyst leads to an increase in the CO_2_ adsorption capacity. Combining the previous analysis of electrochemical performance over the as-prepared catalysts (Fig. 2), we can conclude that regulating the CO_2_ adsorption capacity can control the competing reaction pathways (Fig. 3c). Moderating CO_2_ adsorption is conducive to the adsorption and activation of CO_2_ molecules to participate in the desirable C–N coupling process with 100% carbon efficiency.

**Fig. 4:**
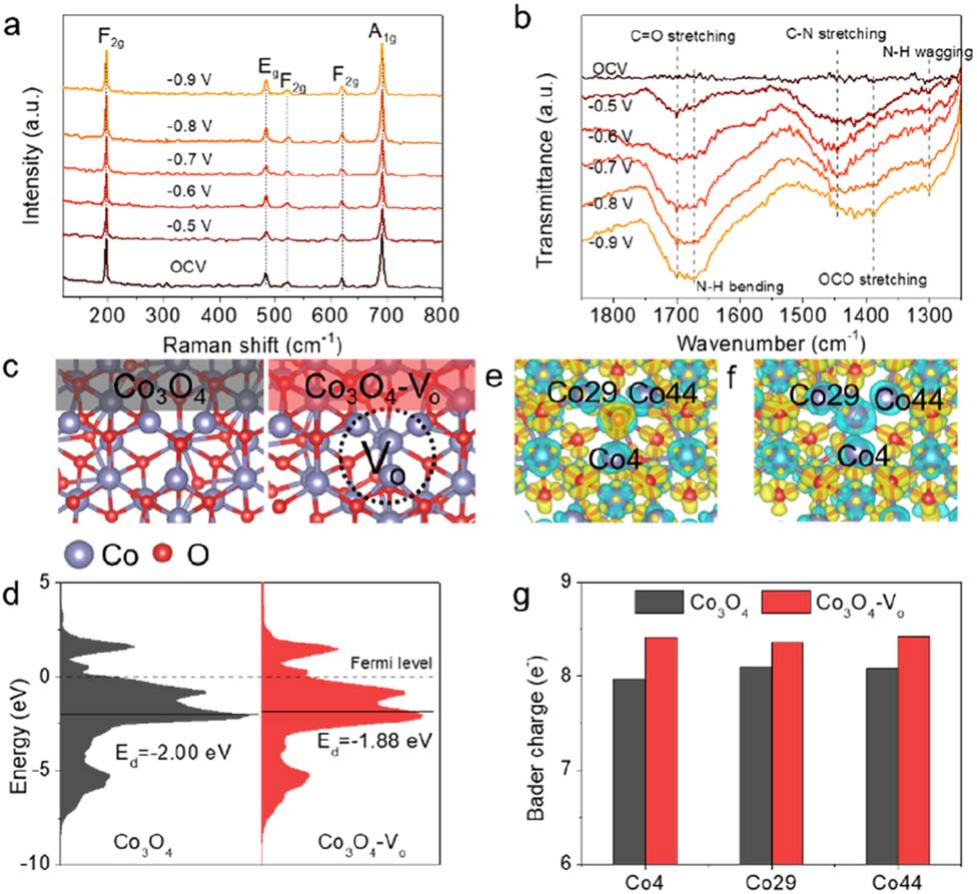
*In situ* Raman (a) and ATR-FTIR (b) spectra recorded at different potentials for Co_3_O_4_-1.0 in CO_2_ saturated 0.2 M KHCO_3_ + 0.02 M KNO_2_ electrolyte. (c) Schematic structures of Co_3_O_4_ and Co_3_O_4_-V_o_. (d) The PDOS of the Co atoms in Co_3_O_4_ and Co_3_O_4_-V_o_ with the d-band center position (*E*_d_) marked by the black line and the Fermi level set as zero. Differential charge densities of (e) Co_3_O_4_ and (f) Co_3_O_4_-V_o_ structures. Yellow and blue contours represent electron accumulation and depletion, respectively. (g) Bader charge comparison of the Co atom around the V_o_ in Co_3_O_4_ and Co_3_O_4_-V_o_.

To gain an in-depth understanding of the C–N coupling mechanism, *in situ* Raman spectroscopy and attenuated total reflection-surface-enhanced Fourier transformed infrared (ATR-FTIR) spectroscopy were carried out. Considering the potential window for urea production, potentials ranging from −0.5 to −0.9 V *vs.* RHE with 0.1 V intervals were selected for spectral acquisition during the *in situ* studies. As shown in Fig. 4a, five distinct peaks were observed in Raman spectra that correspond to the characteristic vibration peaks of Co_3_O_4_.^32^ The intensity of the vibration peak did not change obviously at different applied potentials, suggesting that the Co_3_O_4_ catalyst is stable during electrolysis. However, no reaction intermediate signal was recorded in the Raman spectra, which could be attributed to the strong Raman signal of Co_3_O_4_ that overshadows the faint reaction intermediate signals. Alternatively, some intermediate signals were observed in ATR-FTIR spectra (Fig. 4b). The stretching vibrations observed at around 1700 and 1390 cm^−1^ are attributed to the formation of *CO and *OCO intermediates, respectively,^8,21^ indicating the CO_2_ molecules can adsorb on the surface-active sites of the Co_3_O_4_-1.0 catalyst and further be converted into *CO intermediates. The observation of peaks located at around 1675 and 1300 cm^−1^ corresponded to the bending and wagging modes of the N–H bond, respectively, suggesting the reduction of NO_2_^−^ in the electrolysis.^33^ Specifically, a typical peak at around 1445 cm^−1^ was observed across all tested potentials, demonstrating that the C–N coupling was successfully achieved for urea production.^8,21^ The enhanced peak intensity of the C–N bond at the potential of −0.7 V *vs.* RHE was consistent with the aforementioned experimental results, suggesting that the catalyst exhibited higher activity at this potential.

To strengthen our conclusion, we further conducted density functional theory (DFT) calculations. We built the correlative theoretical models, including Co_3_O_4_ and Co_3_O_4_-V_o_ (Fig. 4c and S25†), based on the above experimental characterizations. The partial density of states (PDOS) calculations on the Co atoms in Co_3_O_4_ and Co_3_O_4_-V_o_ are shown in Fig. 4d. Notably, the d-band center position of Co atoms in the Co_3_O_4_-V_o_ structure (−1.88 eV, relative to the Fermi level) was found to be higher than that in the Co_3_O_4_ structure (−2.00 eV). According to the d-band theory, metal-based catalysts with a higher d-band center (closer to the Fermi level) exhibit stronger interaction between the reaction intermediates and active sites, resulting in an enhanced C–N coupling process. To confirm this assumption, we conducted the simulations on the intermediates involved in CO_2_ and NO_2_^−^ adsorption over the Co_3_O_4_ and Co_3_O_4_-V_o_ structures. The adsorption structures of *CO_2_ and *NO_2_ on Co_3_O_4_ (111) and Co_3_O_4_-V_o_ (111) surfaces are shown in Fig. S26 and S27.† Apparently, the adsorption free energy of *CO_2_ on Co_3_O_4_-V_o_ (−0.31 eV) is lower compared to that on Co_3_O_4_ (−0.17 eV), and the adsorption free energy of *NO_2_ on Co_3_O_4_-V_o_ (−3.39 eV) is also less than that on Co_3_O_4_ (−2.88 eV), indicating that the CO_2_ molecule and NO_2_^−^ ion are more likely to be adsorbed and activated on the Co_3_O_4_-V_o_ surface. In addition, we pursued theoretical insights into the relationship between electronic structure and the electrocatalytic properties to deeply understand the impact of V_o_. To gain further insights into the electronic properties of Co_3_O_4_ and Co_3_O_4_-V_o_, we analyzed the charge transfer within the catalyst structure. As depicted in Fig. 4e and f, we can find that the electrons of Co atoms were consumed in both Co_3_O_4_ and Co_3_O_4_-V_o_, owing to the lower electronegativity of the Co atom compared to the O atom. The detailed Bader charge analysis further revealed that in the Co_3_O_4_-V_o_ structure, the Co4, Co29 and Co44 atoms, which are located in close proximity to the V_o_, had Bader charges of 8.41, 8.37 and 8.43*e*^−^, respectively. These values are higher than those observed in the Co_3_O_4_ structure (Fig. 4g). This indicates that the presence of V_o_ in the Co_3_O_4_ structure leads to the formation of an electron-rich local environment on the Co atoms, which is consistent with the XAS and XPS results. Our computational analysis highlights that the introduction of V_o_ in the catalysts can effectively regulate the electronic structure of the catalyst, leading to the enhancement of adsorption behaviors of intermediates and electrochemical activity for urea synthesis.

## Conclusions

In summary, we achieved urea electrosynthesis by co-reduction of CO_2_ and nitrite using the V_o_-enriched Co_3_O_4_ as the electrocatalyst. The as-prepared Co_3_O_4_-1.0 catalyst demonstrated an outstanding urea yield rate of 3361 mg h^−1^ g_cat_^−1^ with a corresponding FE of 26.1% and 100% carbon selectivity at the potential of −0.7 V *vs.* RHE. The remarkable catalytic performance was attributed to the integration of dual-functional sites in defective Co_3_O_4_, which can be regulated by the amount of V_o_. Experimental measurements and theoretical calculations corroborated the role of V_o_ in constructing the well-matched adsorption/activation sites, which is beneficial for promoting the chemical adsorption of reactants, redistributing the electronic structure of the catalyst. The modulated local environment on Co atoms further enhanced the adsorption of intermediates and decreased the C–N coupling resistance in urea synthesis. Our study demonstrates a route for highly efficient urea synthesis, and the methodology can be used for designing other electrocatalysts for the C–N coupling process.

## Data availability

The data that support the findings of this study are available in the ESI† of this article.

## Author contributions

P. S. L., Q. G. Z., and B. X. H. proposed the project, designed the experiments, and wrote the manuscript. P. S. L. performed all the experiments. P. S. L., J. Y. L., T. B. W., X. N. S., Q. L. M., X. C. K., and X. F. S. performed the analysis of experimental data. Q. G. Z. and B. X. H. co-supervised the whole project. All authors discussed the results and commented on the manuscript.

## Conflicts of interest

There are no conflicts to declare.

## Supplementary Material

SC-015-D3SC06579K-s001

## References

[cit1] Li J., Zhang Y., Kuruvinashetti K., Kornienko N. (2022). Nat. Rev. Chem.

[cit2] Martín A. J., Shinagawa T., Pérez-Ramírez J. (2019). Chem.

[cit3] Xia R., Overa S., Jiao F. (2022). JACS Au.

[cit4] Chen J. G., Crooks R. M., Seefeldt L. C., Bren K. L., Bullock R. M., Darensbourg M. Y., Holland P. L., Hoffman B., Janik M. J., Jones A. K., Kanatzidis M. G., King P., Lancaster K. M., Lymar S. V., Pfromm P., Schneider W. F., Schrock R. R. (2018). Science.

[cit5] Jiang M., Zhu M., Wang M., He Y., Luo X., Wu C., Zhang L., Jin Z. (2023). ACS Nano.

[cit6] Peng X., Zeng L., Wang D., Liu Z., Li Y., Li Z., Yang B., Lei L., Dai L., Hou Y. (2023). Chem. Soc. Rev..

[cit7] Chen C., He N., Wang S. (2021). Small Science.

[cit8] Chen C., Zhu X., Wen X., Zhou Y., Zhou L., Li H., Tao L., Li Q., Du S., Liu T., Yan D., Xie C., Zou Y., Wang Y., Chen R., Huo J., Li Y., Cheng J., Su H., Zhao X., Cheng W., Liu Q., Lin H., Luo J., Chen J., Dong M., Cheng K., Li C., Wang S. (2020). Nat. Chem..

[cit9] Yuan M., Chen J., Bai Y., Liu Z., Zhang J., Zhao T., Wang Q., Li S., He H., Zhang G. (2021). Angew. Chem., Int. Ed..

[cit10] Yuan M., Chen J., Xu Y., Liu R., Zhao T., Zhang J., Ren Z., Liu Z., Streb C., He H., Yang C., Zhang S., Zhang G. (2021). Energy Environ. Sci..

[cit11] Mukherjee J., Paul S., Adalder A., Kapse S., Thapa R., Mandal S., Ghorai B., Sarkar S., Ghorai U. K. (2022). Adv. Funct. Mater..

[cit12] Yuan M., Chen J., Bai Y., Liu Z., Zhang J., Zhao T., Shi Q., Li S., Wang X., Zhang G. (2021). Chem. Sci..

[cit13] Mei Z., Zhou Y., Lv W., Tong S., Yang X., Chen L., Zhang N. (2022). ACS Sustain. Chem. Eng..

[cit14] Qiu M., Zhu X., Bo S., Cheng K., He N., Gu K., Song D., Chen C., Wei X., Wang D., Liu Y., Li S., Tu X., Li Y., Liu Q., Li C., Wang S. (2023). CCS Chem..

[cit15] Sun M., Wu G., Jiang J., Yang Y., Du A., Dai L., Mao X., Qin Q. (2023). Angew. Chem., Int. Ed..

[cit16] Zhang X., Zhu X., Bo S., Chen C., Qiu M., Wei X., He N., Xie C., Chen W., Zheng J., Chen P., Jiang S. P., Li Y., Liu Q., Wang S. (2022). Nat. Commun..

[cit17] Zhang S., Geng J., Zhao Z., Jin M., Li W., Ye Y., Li K., Wang G., Zhang Y., Yin H., Zhang H., Zhao H. (2023). EES Catal..

[cit18] Wang H., Jiang Y., Li S., Gou F., Liu X., Jiang Y., Luo W., Shen W., He R., Li M. (2022). Appl. Catal. B Environ..

[cit19] Qin J., Liu N., Chen L., Wu K., Zhao Q., Liu B., Ye Z. (2022). ACS Sustain. Chem. Eng..

[cit20] Meng N., Ma X., Wang C., Wang Y., Yang R., Shao J., Huang Y., Xu Y., Zhang B., Yu Y. (2022). ACS Nano.

[cit21] Lv C., Zhong L., Liu H., Fang Z., Yan C., Chen M., Kong Y., Lee C., Liu D., Li S., Liu J., Song L., Chen G., Yan Q., Yu G. (2021). Nat. Sustain..

[cit22] Wei X., Liu Y., Zhu X., Bo S., Xiao L., Chen C., Nga T. T. T., He Y., Qiu M., Xie C., Wang D., Liu Q., Dong F., Dong C.-L., Fu X.-Z., Wang S. (2023). Adv. Mater..

[cit23] Cao N., Quan Y., Guan A., Yang C., Ji Y., Zhang L., Zheng G. (2020). J. Colloid Interface Sci..

[cit24] Feng Y., Yang H., Zhang Y., Huang X., Li L., Cheng T., Shao Q. (2020). Nano Lett..

[cit25] Zhu Y., Wang J., Koketsu T., Kroschel M., Chen J.-M., Hsu S.-Y., Henkelman G., Hu Z., Strasser P., Ma J. (2022). Nat. Commun..

[cit26] Cai Z., Bi Y., Hu E., Liu W., Dwarica N., Tian Y., Li X., Kuang Y., Li Y., Yang X.-Q., Wang H., Sun X. (2018). Adv. Energy Mater..

[cit27] Martínez-Arias A., Conesa J. C., Soria J. (2007). Res. Chem. Intermed..

[cit28] Schmitt R., Nenning A., Kraynis O., Korobko R., Frenkel A. I., Lubomirsky I., Haile S. M., Rupp J. L. M. (2020). Chem. Soc. Rev..

[cit29] Kim J. H., Jang Y. J., Kim J. H., Jang J.-W., Choi S. H., Lee J. S. (2015). Nanoscale.

[cit30] Tan H., Tang B., Lu Y., Ji Q., Lv L., Duan H., Li N., Wang Y., Feng S., Li Z., Wang C., Hu F., Sun Z., Yan W. (2022). Nat. Commun..

[cit31] Pan L., Wang J., Lu F., Liu Q., Gao Y., Wang Y., Jiang J., Sun C., Wang J., Wang X. (2023). Angew. Chem., Int. Ed..

[cit32] Wang Y., Wei X., Hu X., Zhou W., Zhao Y. (2019). Catal. Lett..

[cit33] Geng J., Ji S., Jin M., Zhang C., Xu M., Wang G., Liang C., Zhang H. (2023). Angew. Chem., Int. Ed..

