# Supplementary material for: Efficient C–N coupling for urea electrosynthesis on defective Co3O4 with dual-functional sites
Source: Chem Sci. 2024 Jan 18;15(9):3233–9. doi: 10.1039/d3sc06579k (PMC10901497; doi:10.1039/d3sc06579k)
Supplement: SC-015-D3SC06579K-s001 [file SC-015-D3SC06579K-s001.pdf]

Electronic Supplementary Material (ESI) for Chemical Science.
This journal is © The Royal Society of Chemistry 2024

Electronic Supplementary Material (ESI) for

Efficient C-N coupling for urea electrosynthesis on defective Co_3O_4 with dual-functional sites

Methods

Chemicals

$Co(NO_3)_2 \cdot 6H_2O$ (ACS, >98%) and sodium citrate ($C_6H_5Na_3O_7$, 99%) were purchased from Alfa. NaCl (AR, 99.5 %), $KHCO_3$ (AR, 99.5%), NaOH (AR, 96%), NH_4Cl (99.99%), urease (≥45 units/mg dry weight), sodium nitroferricyanide (III) dihydrate ($C_5FeN_6Na_2O \cdot 2H_2O$, AR, 99%) and KNO_2 (AR, 97%) were provided by Aladdin. Salicylic acid ($C_7H_6O_3$, 99%), and NaClO solution (10%) were obtained from Innochem (Beijing) Technology Co.,Ltd. Carbon fiber paper was obtained from TORAY. All chemicals were analytical grade and used as received without further purification. All electrolyte solutions were prepared using Milli-Q water (18.2 MΩ cm).

Preparation of Co_3O_4 catalysts

In a typical synthesis, 0.582 g $Co(NO_3)_2 \cdot 6H_2O$ and 0.8 g NaCl were mixed in an agate mortar for 30 min. Then, 1 mL NaOH solution (5 mmol L^{-1}) was added into the evenly mixed precursors and ground for 10 min. Afterwards, the resulting solution was dried at 80°C in an oven under air atmosphere for 24 h, yielding a mauve solid. The mauve solid obtained was ground with a mortar and pestle for 20 min, then transferred into a ceramic crucible and heated up in a tube furnace to 450 °C (with a ramp rate of 5 °C min^{-1}) under an Ar gas flow of 20 standard cubic centimeters per minute (sccm) and maintained for 6 h. Afterwards, the calcined sample was washed by deionized water three times and dried overnight in a vacuum oven at 80 °C to obtain the pristine Co_3O_4 product. The Co_3O_4 with different amounts of oxygen vacancy were synthesized via thermal treatment under H_2/Ar (10%/90%, v/v) mixture atmosphere at 200 °C for different times (0.5, 1.0, and 1.5 h), named as Co_3O_4-0.5, Co_3O_4-1.0, and Co_3O_4-1.5, respectively.

Material characterization

The morphologies of materials were characterized by a HITACHI S-4800 scanning electron microscope (SEM) and a JEOL JEM-2100F high-resolution transmission electron microscopy (HRTEM). Powder X-ray diffraction (XRD) patterns were acquired from an X-ray diffractometer (Model D/MAX2500, Rigaka) with Cu-Kα radiation, and the scan speed was 5° min^{-1}. The electron paramagnetic resonance (EPR) was performed on a JEOL JES-FA200 spectrometer at 298 K with 4.00 G modulation amplitude and a magnetic field modulation of 100 kHz. X-ray photoelectron spectroscopy (XPS) analysis was conducted on the Thermo Scientific ESCALab 250Xi (USA) using 200 W monochromatic Al Kα radiation. The 500 μm X-ray spot was used for XPS analysis. The base pressure in the analysis chamber was about 3×10^{-10} mbar. Temperature programmed desorption (TPD) was conducted on the Micromeritics AutoChem 2950 HP chemisorption analyzer. The X-ray absorption spectroscopy (XAS) measurements were performed at 1W1B beamline station of Beijing Synchrotron Radiation Facility (BSRF) and BL14W1 beamline station of Shanghai Synchrotron Radiation Facility (SSRF). The energy was tuned by Si (111) monochromator. The data were collected in fluorescence

excitation mode using a Lytle detector.

Preparation of cathode electrode

The catalyst ink was prepared by ultrasonic dispersion of 1 mg of the catalyst powder with 5 μL Nafion solution (5 wt %) in 1 mL acetone for 30 min. Next, the as-prepared ink was drop-coated on a carbon fiber paper (1 x 0.5 cm^2) achieving the catalyst loading of 0.5 mg cm^{-2}. The electrode was then dried in the atmosphere for the subsequent electrochemical testing experiments.

Electrochemical measurements

Electrochemical studies were conducted in an electrochemical H-cell separated by a Nafion 117 membrane (Alfa). The saturated Ag/AgCl electrode and Pt foam were used as the reference and counter electrodes, respectively. The electrolysis was conducted using a CHI 660e electrochemical workstation. The Ar saturated 0.1 M $KHCO_3$ + 0.02 M KNO_2, CO_2 saturated 0.1 M $KHCO_3$ and CO_2 saturated 0.1 M $KHCO_3$ + 0.02 M KNO_2 were used as the cathode electrolyte for nitrite electroreduction, CO_2 reduction reaction, and urea electrosynthesis, respectively. 0.1 M $KHCO_3$ aqueous solution was used as the anodic electrolyte. Electrochemical CO_2 reduction reaction and urea electrosynthesis were carried out with CO_2 bubbling. All potentials were converted to the reversible hydrogen electrode (RHE) reference scale using the relation $E_{RHE} = E_{Ag/AgCl} + 0.197 + 0.059 \times pH$ and compensated with the solution resistance. Controlled potential electrolysis was then performed at each potential ranging from -0.5 to -0.9 V vs. RHE for 20 min.

Gaseous and liquid products analysis

The gaseous product in the electrochemical experiment was collected by using a gas bag and analyzed by gas chromatography (GC, HP 4890D). The NH_3 was quantified using the indophenol blue method using UV-vis spectrophotometry. Briefly, electrolyte (400 μL, pipetted from the cathodic chamber), coloring solution (2 mL) containing salicylic acid (5 wt%) + sodium citrate (5 wt%) + NaOH (0.75 M), oxidizing solution containing NaClO (1 mL, 0.05 M), and sodium nitroferricyanide (III) dihydrate solution (200 uL, 1 wt%) were added in turn and mixed in a sample tube, and the above solution was then diluted to 10 mL with fresh electrolyte. The UV-vis measurements were performed within a range of 550 to 800 nm after the solution was left in the dark at room temperature for 2 h. The maximum UV-vis absorption peak was obtained at 665 nm. The concentration absorbance curve was calibrated using the standard NH_4Cl solution with different concentrations (0.5, 1.0, 1.5, 2.0, 2.5 μmmol/mL) in 0.1 M $KHCO_3$ + 0.02 M KNO_2 solution and measuring the absorbance at 665 nm of the samples. A fitting curve (y =0.795x, R^2 = 0.9996) with ideal linear relation was obtained. Urea was decomposed by urease (C_{urease} = 5 mg/mL; V_{urea}/V_{urease} = 10/1) into CO_2 and two ammonia molecules at 37°C for 30 min. After the decomposition, NH_3 concentration of urea electrolyte with urease was detected via above indophenol blue method. At the same time, NH_3 moles (m_{NH3}) contained in urea electrolyte without urease was also quantified by same indophenol blue method. The total moles (m_{urease}) of NH_3 in the electrolyte were measured by the UV-vis spectrophotometry and shown as $2m_{urea}+m_{NH3}$, where $2m_{urea}$ represents the moles of ammonia coming from the decomposition. Therefore, the moles of urea (m_{urea}) produced were calculated by $(m_{urease}- m_{NH3})/2$.

The Faradaic efficiency (FE) of the product is:

$$FE = \frac{m}{Q / NF} \times 100\%$$

Where Q is the charge (C), F is the Faradaic constant (96485 C mol^{-1}), N is the number of electrons required to generate the product, and m is the moles of products. For the H_2, NH_3, and urea, the N is 2, 6, and 12, respectively.

Double layer capacitance (C_{dl}) measurement

The cyclic voltammetry measurement was conducted using a H-cell, and the other conditions were the same as that of the CO_2 reduction. Cyclic voltammogram measurements of the catalysts were conducted from -0.34 to -0.44 V versus Ag/AgCl with various scan rates to obtain the double layer capacitance (C_{dl}) of different catalysts. The C_{dl} was estimated by plotting the Δj (j_a-j_c) at -0.39 V versus Ag/AgCl against the scan rates, in which j_a and j_c are the anodic and cathodic current densities, respectively. The linear slope was equivalent to twice C_{dl}.

^{15}N isotope labeling experiment

The ^{15}N isotopic labeling experiments were conducted using 0.2 M $KHCO_3$ + 0.02 M $K^{15}NO_2$ (99 atom %) electrolytes with CO_2 as feeding gas. After the potentiostatic electrolysis at -0.7 V (vs. RHE) for 4 h, the electrolyte was concentrated at 60 °C. The ^1H NMR spectra were measured on a Bruker Avance III 400 HD spectrometer. The DMSO-d_6 was used as the internal standard.

In situ Raman measurements

In situ Raman measurements were carried out using a Horiba LabRAM HR Evolution Raman microscope in a modified H-cell, which was produced by GaossUnion (Tianjin) Photoelectric Technology Company. The carbon paper loaded with catalyst was used as working electrode, a saturated Ag/AgCl electrode and Pt wire were used as reference electrode and counter electrode, respectively. CO_2 saturated 0.1 M $KHCO_3$ + 0.02 M KNO_2 aqueous solution and 0.1 M $KHCO_3$ aqueous solution were used as electrolyte and circulated through the cathodic chamber and anodic chamber, respectively, by peristaltic pumps at a rate of 5 mL min^{-1}. A 785 nm excitation laser was used and signals were recorded using a 20 s integration and by averaging two scans. The signals were recorded at different applied potentials, and a 10 min electrolysis was conducted to gain the steady state before the collection of Raman spectra.

In situ ATR-FTIRS measurements

A Nicolet 6700 FT-IR equipped with a mercury cadmium telluride detector cooled with liquid nitrogen was employed in the in-situ electrochemical study. The measurement was conducted in a modified electrochemical cell, the catalyst was dropped on the silicon ATR crystal deposited with Au film, which was used as working electrode. The Pt wire and saturated Ag/AgCl electrode were used as counter electrode and reference electrode, respectively. CO_2 saturated 0.1 M $KHCO_3$ + 0.02 M KNO_2 aqueous solution and 0.1 M $KHCO_3$ aqueous solution were used as electrolyte and circulated through the cathodic chamber and anodic chamber, respectively, by peristaltic pumps at a rate of 5 mL min^{-1}. The signals were recorded at different applied potentials, and a 10 min electrolysis was conducted to gain the steady state before the collection of IR spectra.

Theoretical calculations

All the calculations were performed in the framework of the density functional theory with

the projector augmented plane-wave (PAW) method using the Vienna ab initio simulation package (VASP).[1] The generalized gradient approximation proposed by Perdew, Burke, and Ernzerh was selected for the exchange-correlation potential.[2] The Grimme's DFT-D3 correction method[3] was included to describe the weak dispersion interactions during surface adsorption. The cut-off energy for the plane wave was set to 500 eV. The energy criterion was set to 10^{-5} eV in the iterative solution of the Kohn-Sham equation. A vacuum layer of 15 Å was added perpendicular to the sheet to avoid artificial interaction between periodic images. A 2×2×1 Monkhorst-Pack k-point sampling was set for all models. U (Co_{3d}) value of 3.5 eV was applied to the Co_{3d} state. All the structures were relaxed until the residual forces on the atoms had declined to less than 0.02 eV $Å^{-1}$. The free energy change (ΔG) of each elementary reaction can be computed by the following equation:

$$\Delta G = \Delta E + \Delta ZPE - T\Delta S,$$

where ΔE, ΔZPE, T, and ΔS are the reaction energy difference, zero-point energy change, temperature, and entropy change, respectively.

Figures and Table

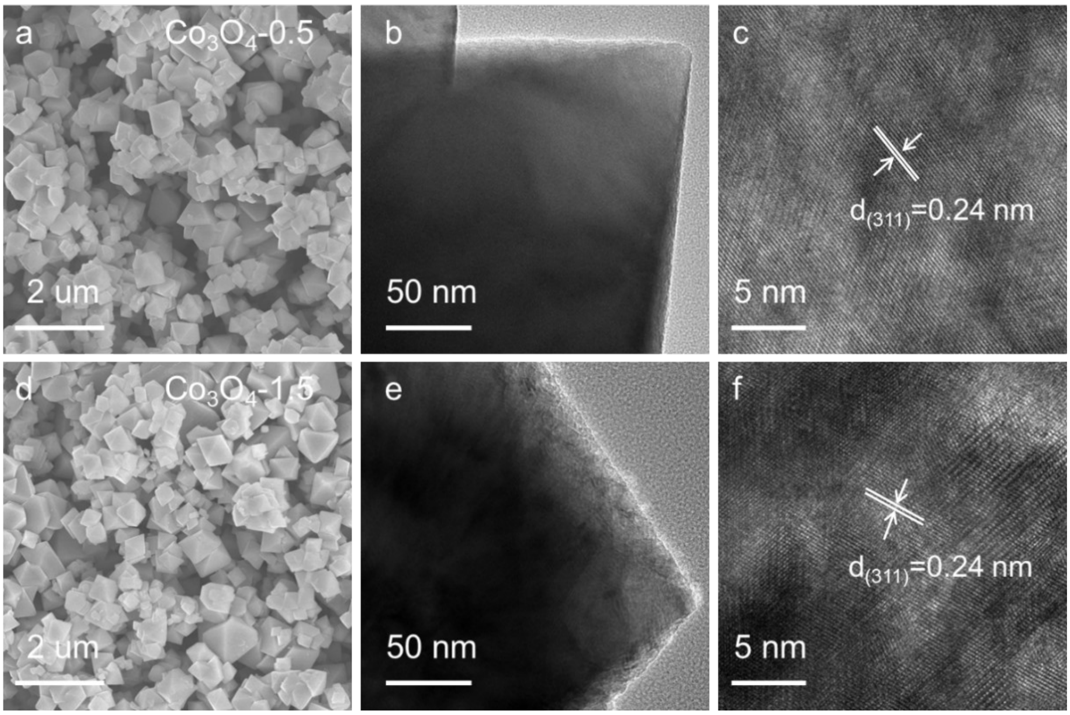

Figure S1. SEM, TEM and HRTEM images of (a, b, c) Co_3O_4-0.5 and (d, e, f) Co_3O_4-1.5.

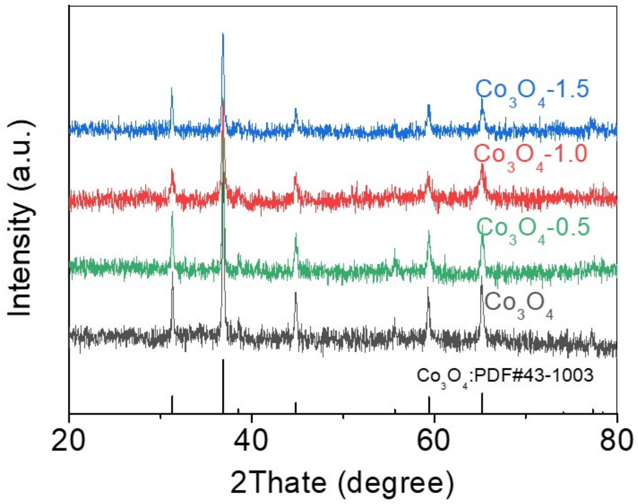

Figure S2. XRD patterns of pristine Co_3O_4, Co_3O_4-0.5, Co_3O_4-1.0, and Co_3O_4-1.5.

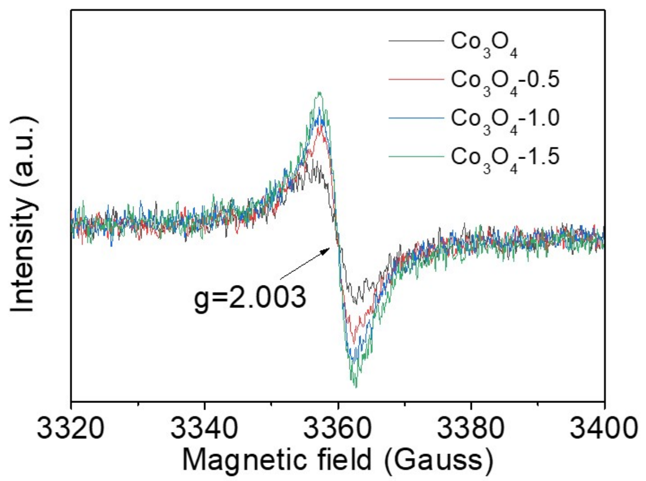

Figure S3. EPR spectra of Co_3O_4, Co_3O_4-0.5, Co_3O_4-1.0 and Co_3O_4-1.5.

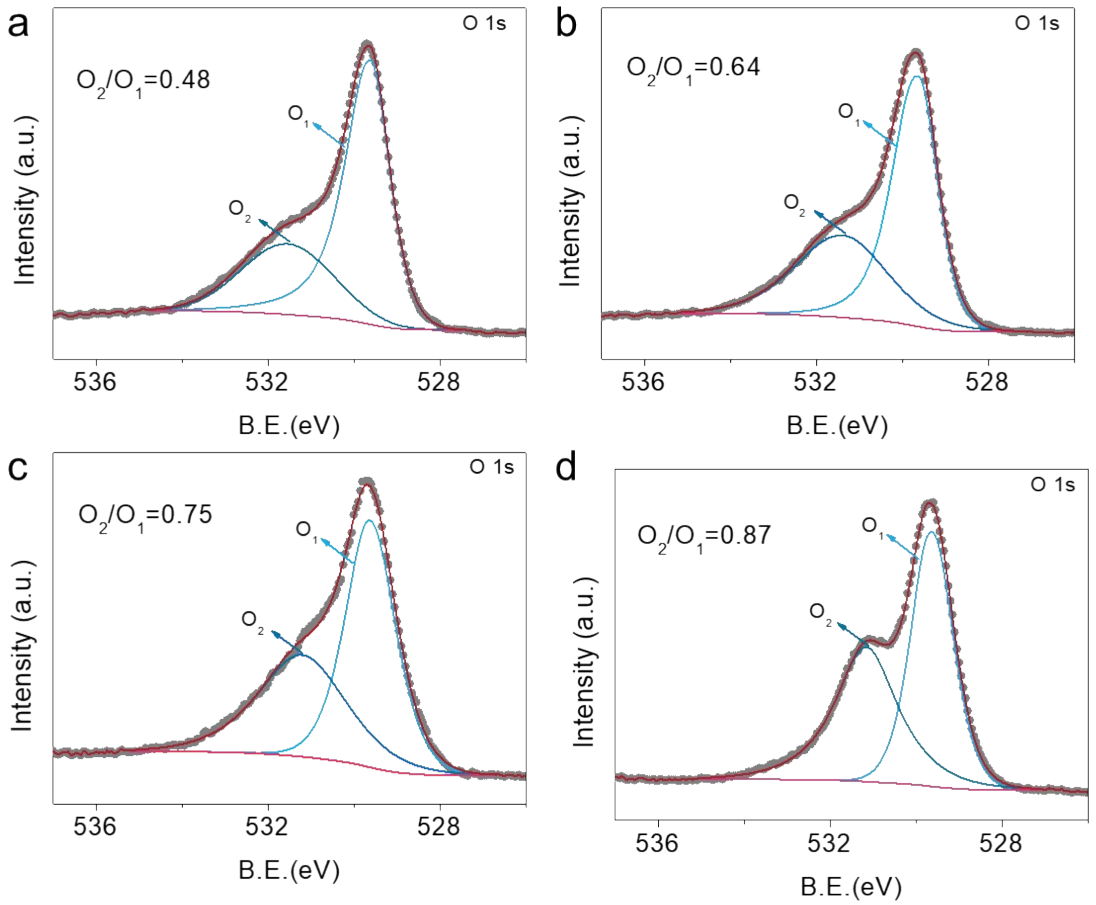

Figure S4. XPS spectra of O1s in (a) pristine Co_3O_4, (b) Co_3O_4-0.5, (c) Co_3O_4-1.0, and (d) Co_3O_4-1.5. The O_2/O_1 ratio means the ratio of oxygen vacancies (O_2) and lattice oxygen (O_1) area in the O1s spectra.

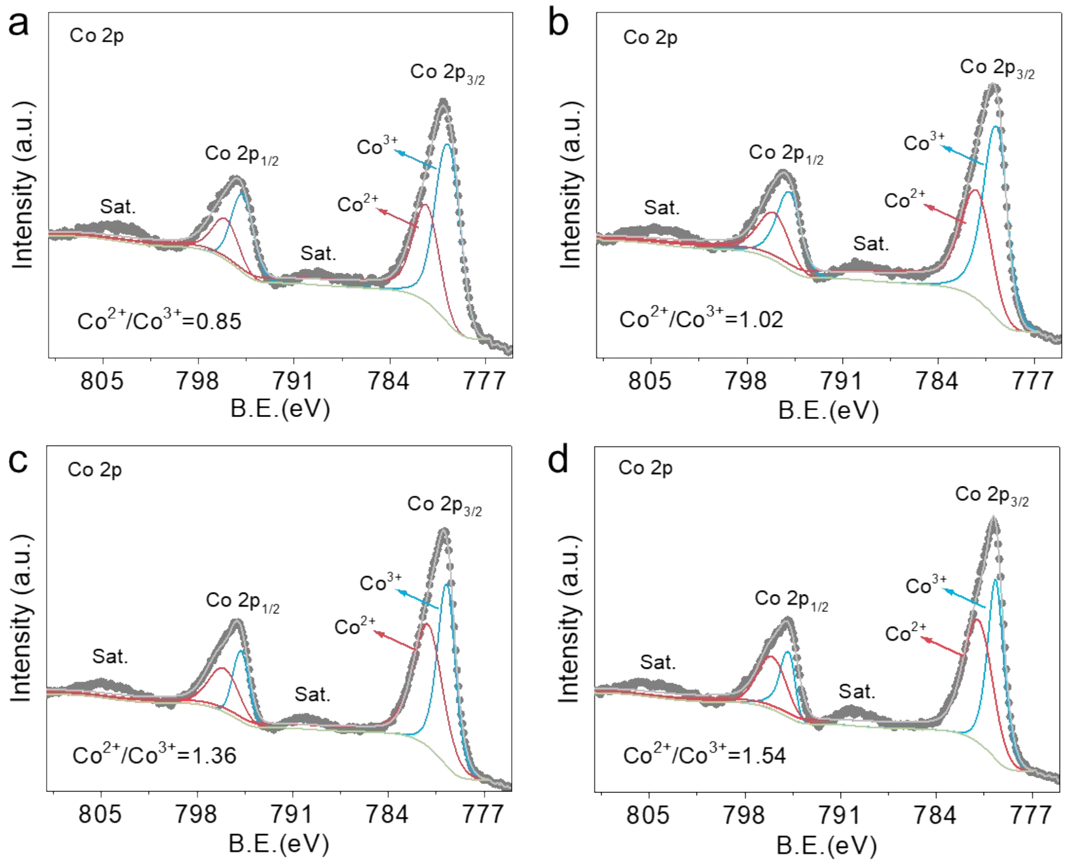

Figure S5. XPS spectra of Co2p in (a) pristine Co_3O_4, (b) Co_3O_4-0.5, (c) Co_3O_4-1.0, and (d) Co_3O_4-1.5. The Co^{2+}/Co^{3+} ratio means the ratio of Co^{2+} and Co^{3+} area in the Co2p spectra.

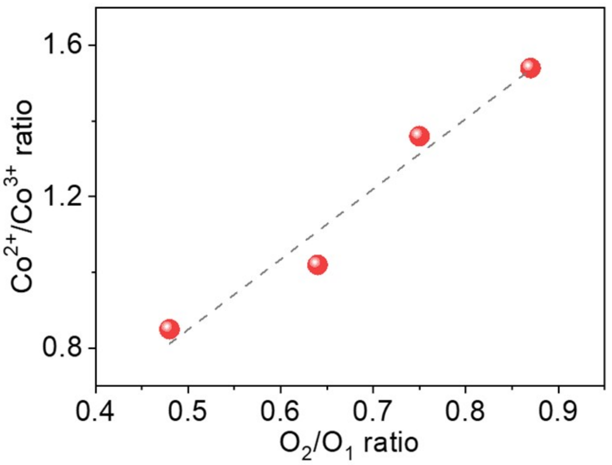

Figure S6. The ratio of Co^{2+}/Co^{3+} vs. the ratio of O_2/O_1 in the as-prepared catalysts based on XPS semiquantitative analysis.

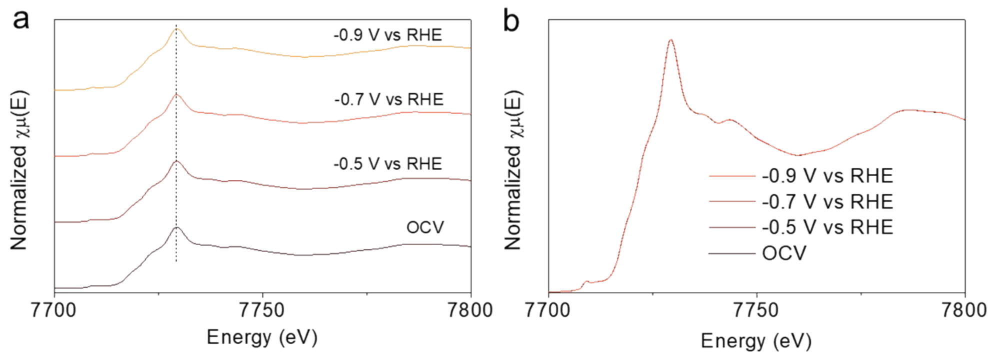

Figure S7. Operando XANES under the electrochemical condition of Co K-edge over Co_3O_4-1.0. OCV represents open-circuit voltage.

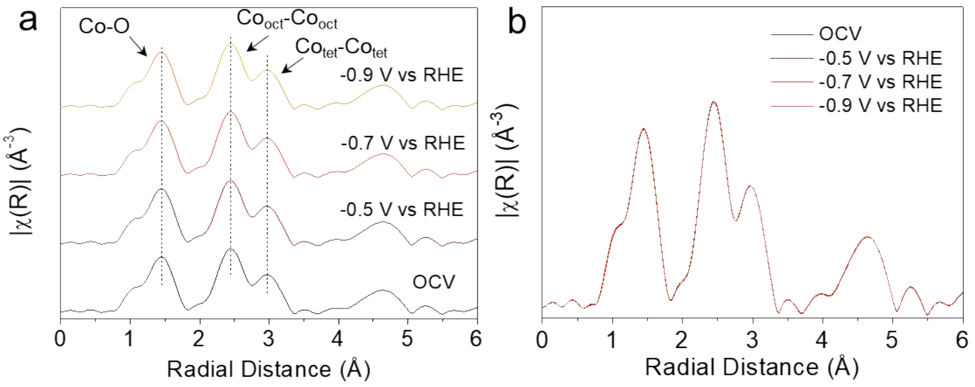

Figure S8. Operando flourier-transformed EXAFS spectra under the electrochemical condition of Co K-edge over Co_3O_4-1.0. OCV represents open-circuit voltage.

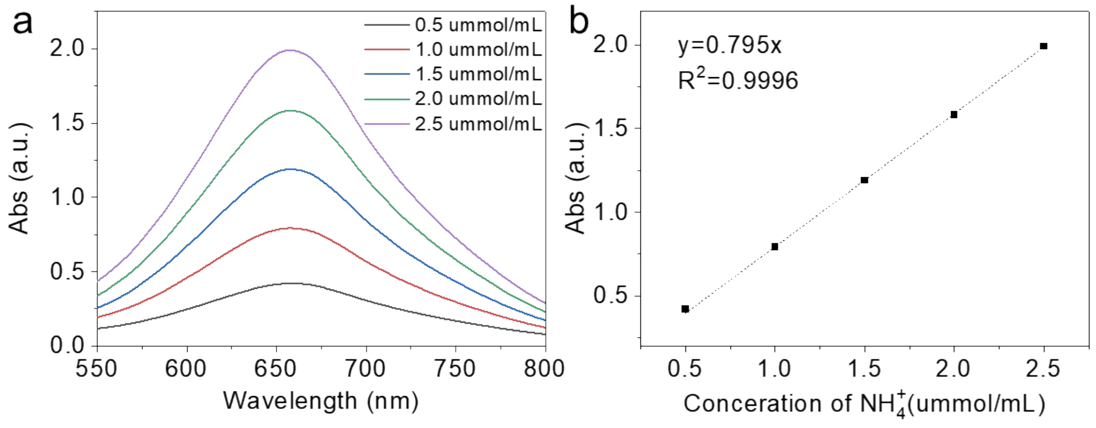

Figure S9. (a) UV-vis absorption spectra of NH_4^+ solutions with different concentrations. (b) Standard calibration curve used for quantifying NH_4^+.

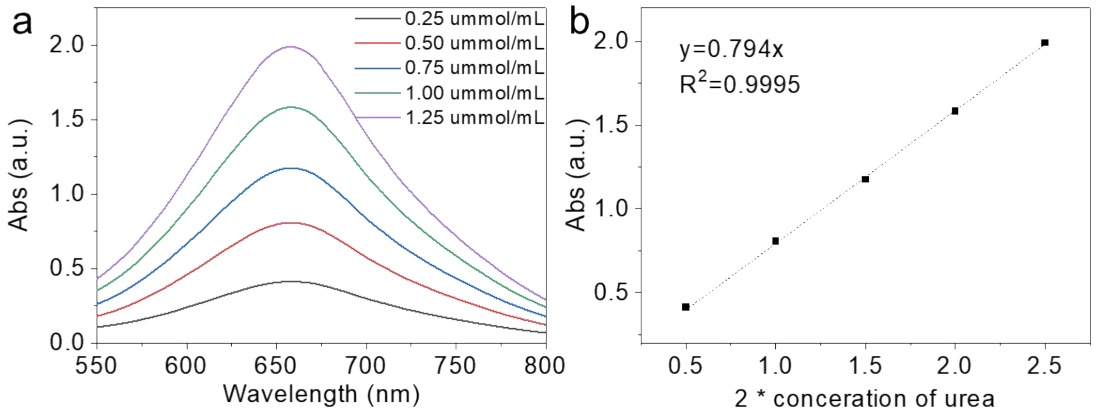

Figure S10. (a) UV-vis absorption spectra of urea solutions with different concentrations after decomposition by urease. (b) The calibration curve used for quantifying urea.

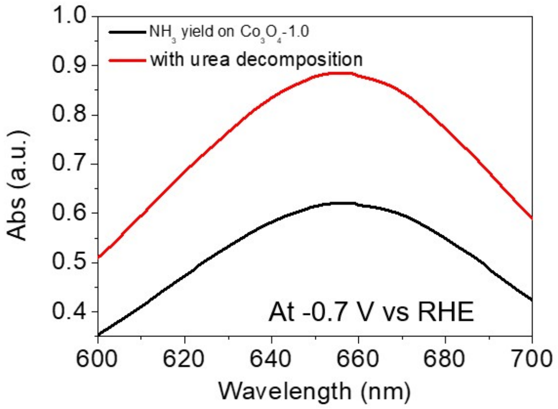

Figure S11. Typical UV-vis absorption spectra for NH_3 and urea quantification.

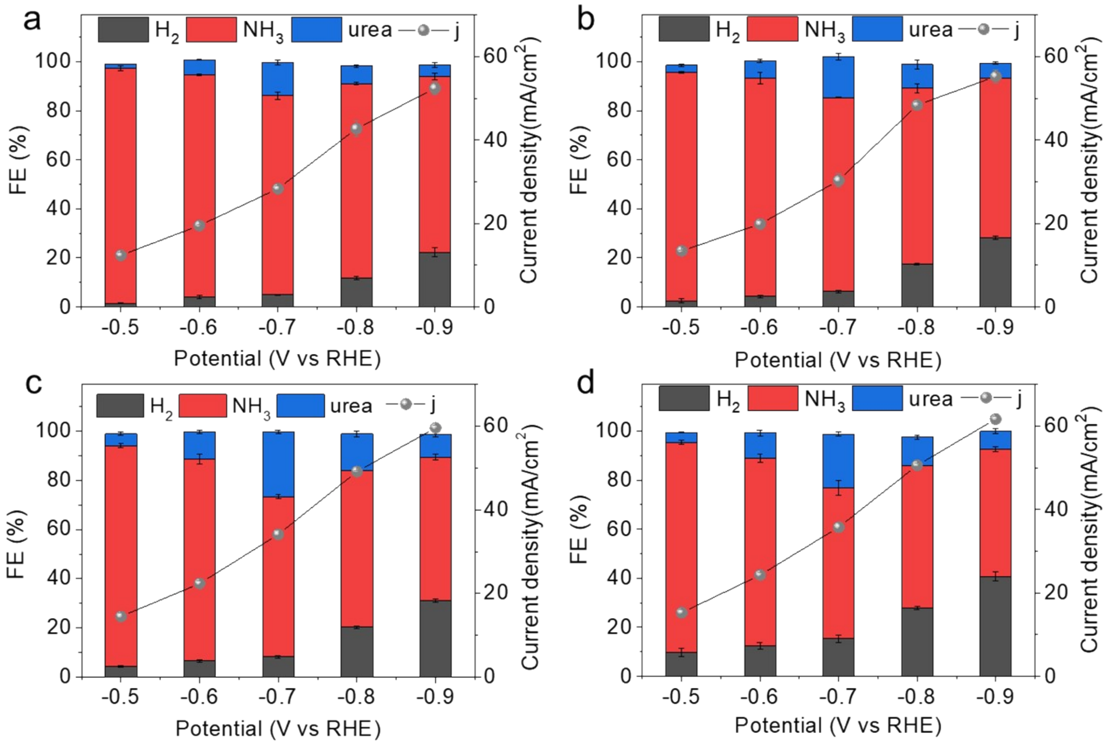

Figure S12. The FEs of major products in CO_2 saturated 0.2 M $KHCO_3$ + 0.02 M KNO_2 enabled by (a) pristine Co_3O_4, (b) Co_3O_4-0.5, (c) Co_3O_4-1.0, and (d) Co_3O_4-1.5. electrocatalyst under different potentials. Error bars represent standard deviations from three repeated measurements.

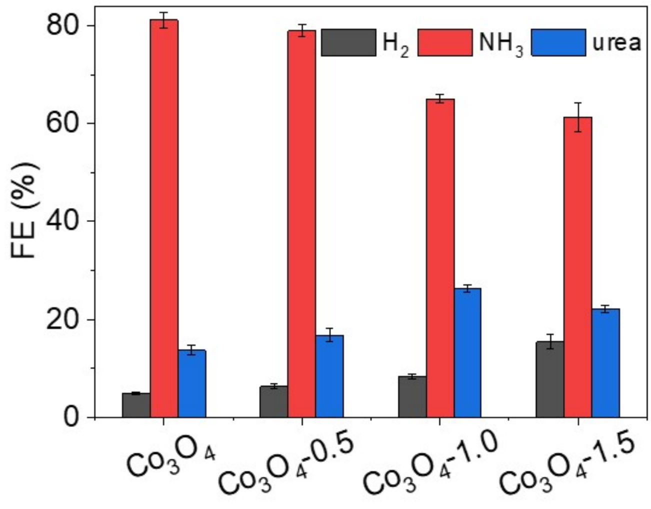

Figure S13. FEs of H_2, NH_3 and urea at the potential of -0.7 V (vs. RHE) over pristine Co_3O_4, Co_3O_4-0.5, Co_3O_4-1.0, and Co_3O_4-1.5 in CO_2 saturated 0.2 M $KHCO_3$ + 0.02 M KNO_2 electrolyte.

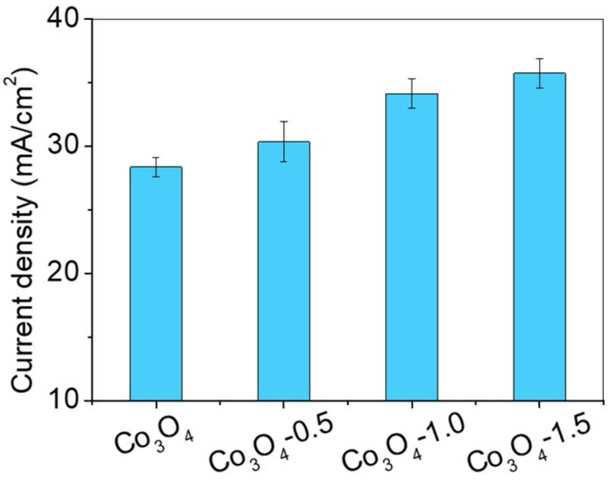

Figure S14. The total current density comparison over pristine Co_3O_4, Co_3O_4-0.5, Co_3O_4-1.0, and Co_3O_4-1.5 in CO_2 saturated 0.2 M $KHCO_3$ + 0.02 M KNO_2 electrolyte at the potential of -0.7 V vs. RHE.

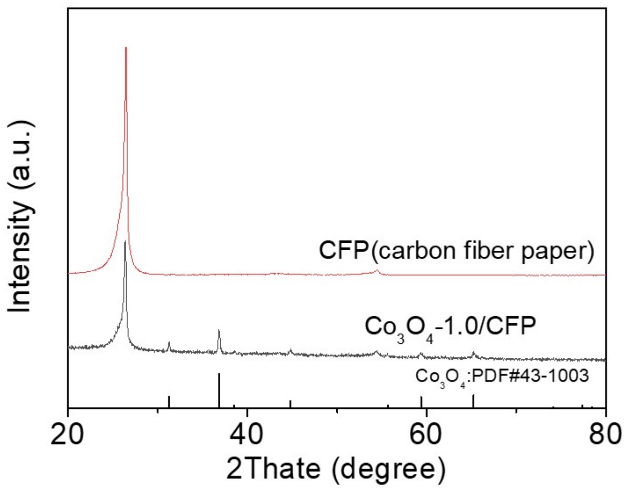

Figure S15. XRD patterns of CFP (carbon fiber paper) and Co_3O_4-1.0/CFP after long-term stability test.

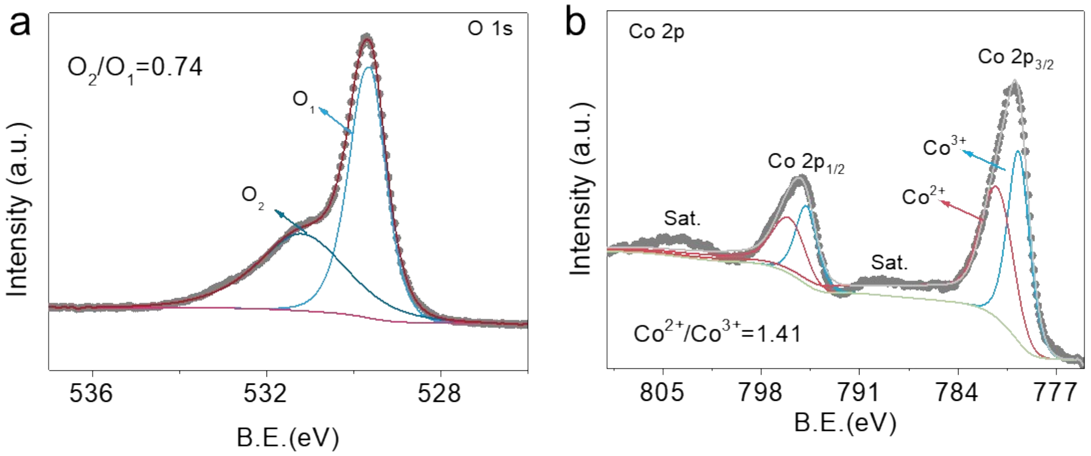

Figure S16. XPS spectra of (a) O1s and (b) Co2p in Co_3O_4-1.0 after long-term stability test.

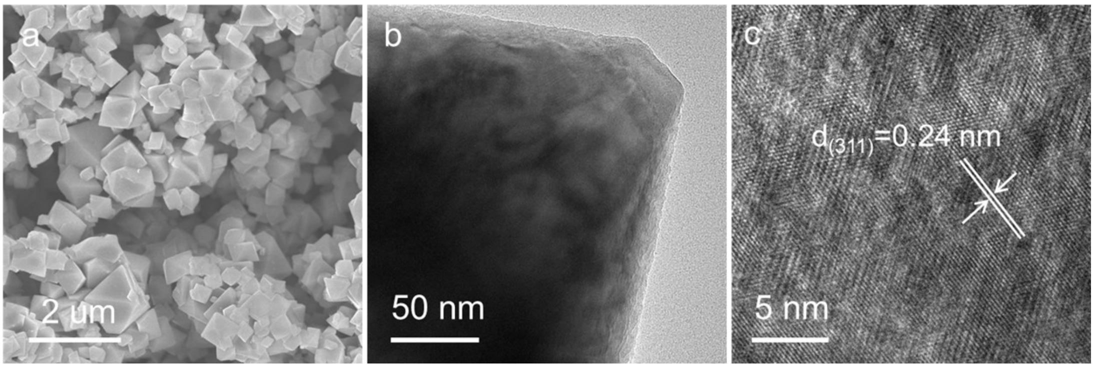

Figure S17. (a) SEM, (b) TEM and (c) HRTEM images of Co_3O_4-1.0 after long-term stability test.

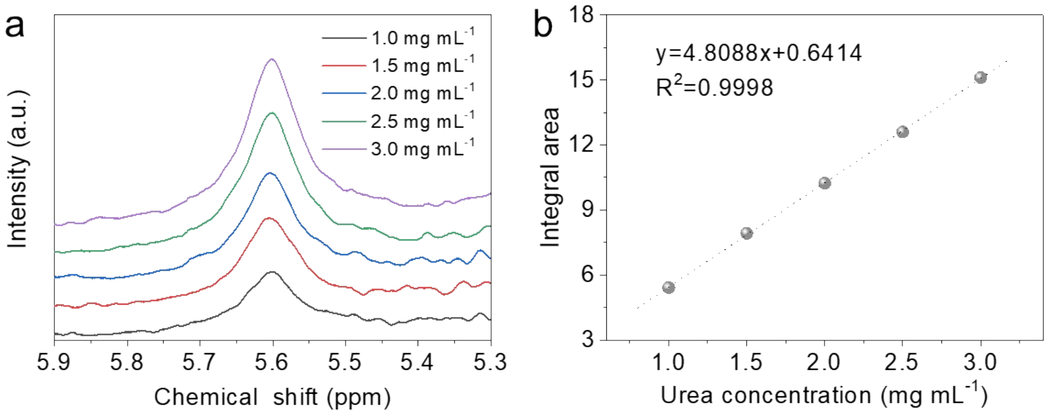

Figure S18. (a) ^1H NMR spectra of standard urea solution with various concentrations of 1.0-3.0 mg mL^{-1}. (b) The calibration curve used for quantifying urea.

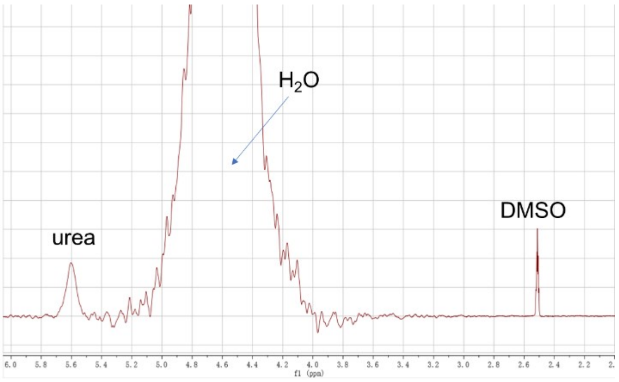

Figure S19. Typical ^1H NMR spectra of acquired liquid samples after electrosynthesis using Co_3O_4-1.0 as the catalyst in CO_2 saturated 0.2 M $KHCO_3$ + 0.02 M KNO_2 electrolyte.

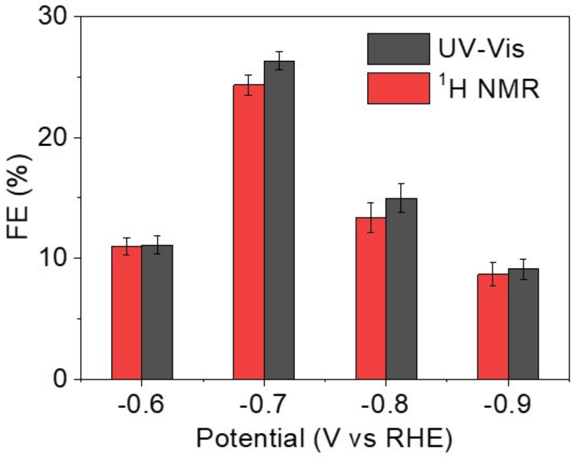

Figure S20. The FEs of urea over Co_3O_4-1.0 electrocatalyst under different potentials detected by the UV-vis method and ^1H NMR method.

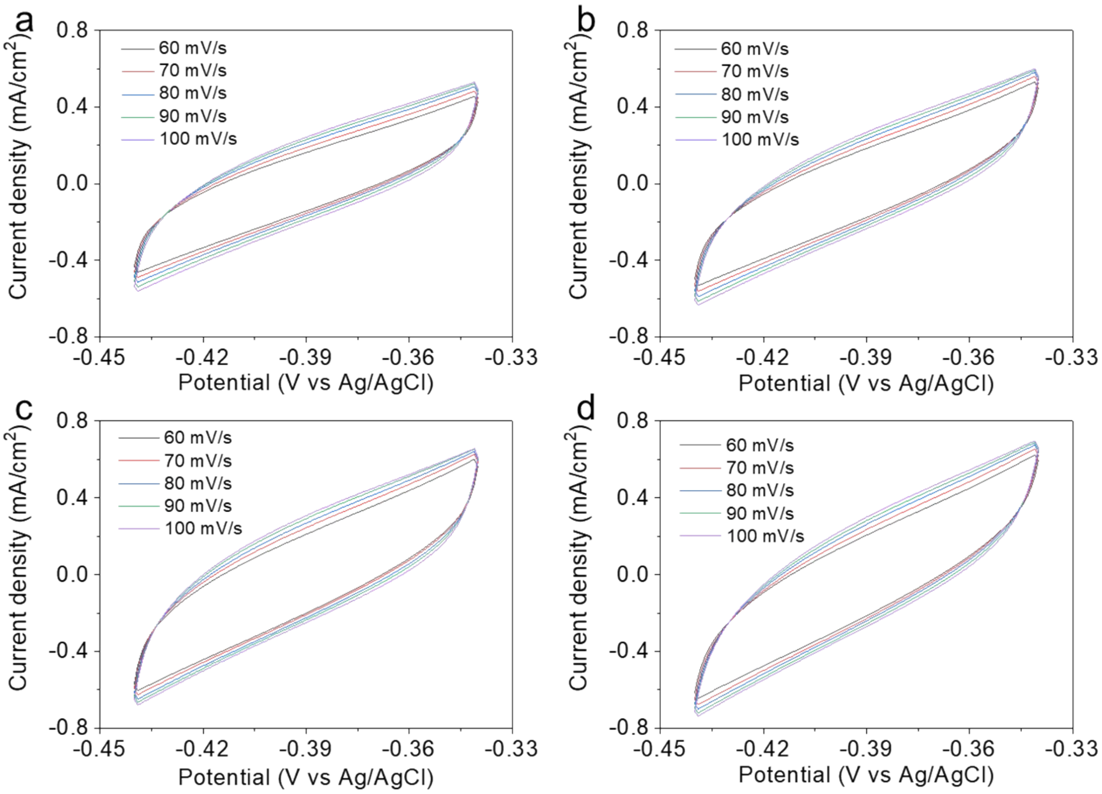

Figure S21. Electric double layer capacitance (C_{dl}) measurements at the non-Faradaic region (from -0.34 to -0.44 V vs. Ag/AgCl) with various scan rates (60 mV s^{-1}-100 mV s^{-1}) of (a) pristine Co_3O_4, (b) Co_3O_4-0.5, (c) Co_3O_4-1.0, and (d) Co_3O_4-1.5.

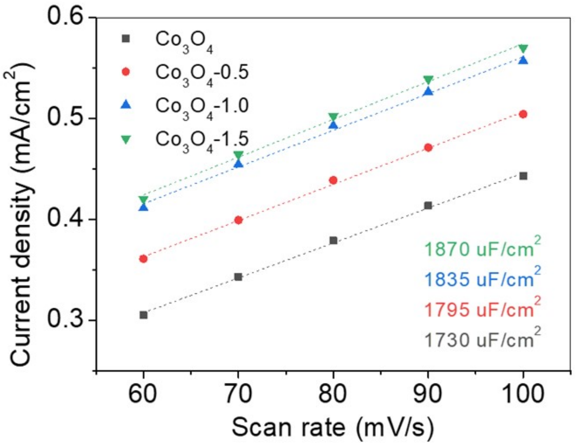

Figure S22. Charging current density differences plotted against scan rates over pristine Co_3O_4, Co_3O_4-0.5, Co_3O_4-1.0, and Co_3O_4-1.5.

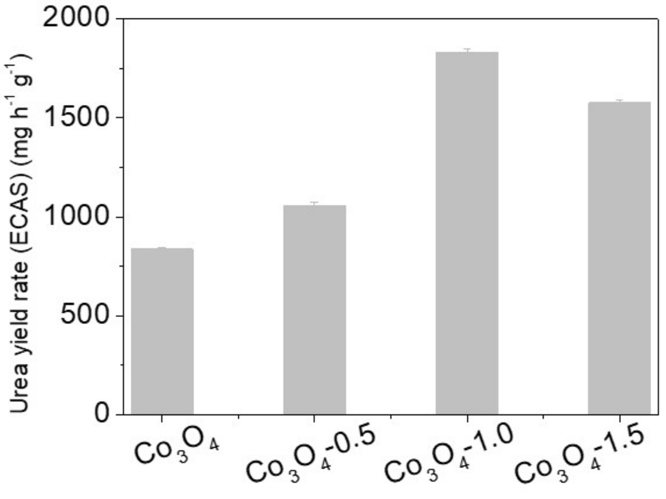

Figure S23. The urea yield rate normalized to ECAS (mF/cm^2) at the potential of -0.7 V (vs. RHE) over pristine Co_3O_4, Co_3O_4-0.5, Co_3O_4-1.0, and Co_3O_4-1.5.

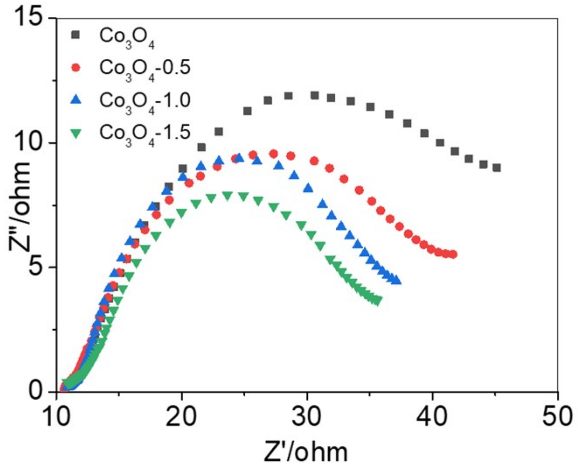

Figure S24. Electrochemical impedance spectroscopy curves of pristine Co_3O_4, Co_3O_4-0.5, Co_3O_4-1.0, and Co_3O_4-1.5.

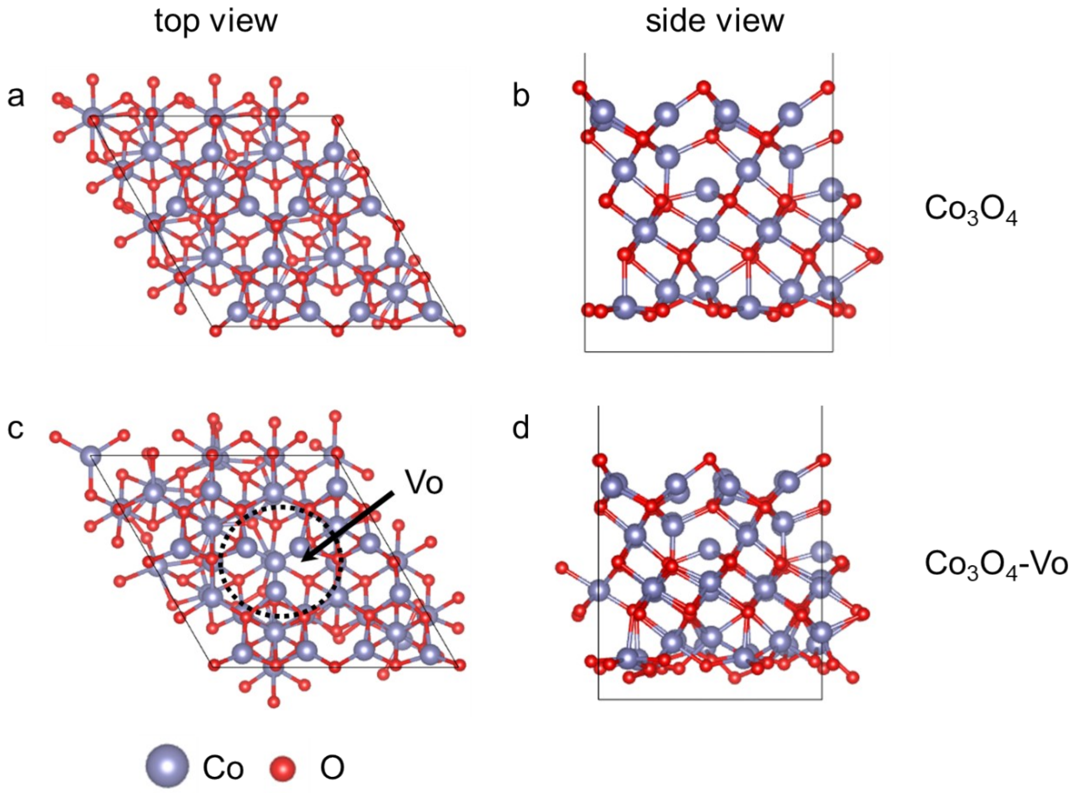

Figure S25. Schematic structures of (a, b) Co_3O_4 and (c, d) Co_3O_4-V_o with top and side view.

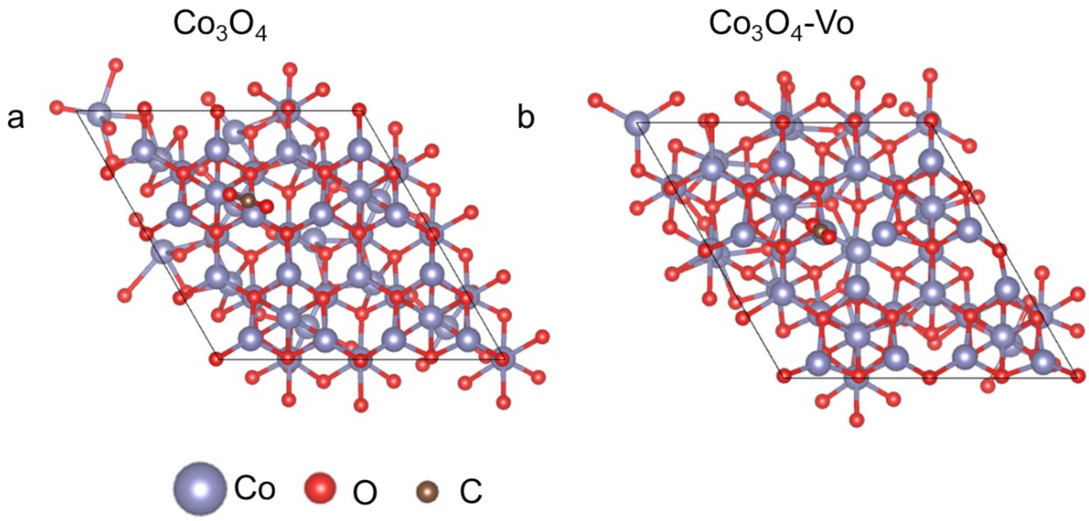

Figure S26. The adsorption structures of $*CO_2$ on (a) Co_3O_4 (111) and (b) Co_3O_4-V_o (111) surface. The adsorption energy of CO_2 over Co_3O_4 (111) and (b) Co_3O_4-V_o (111) surface was -0.17 eV and -0.31 eV, respectively.

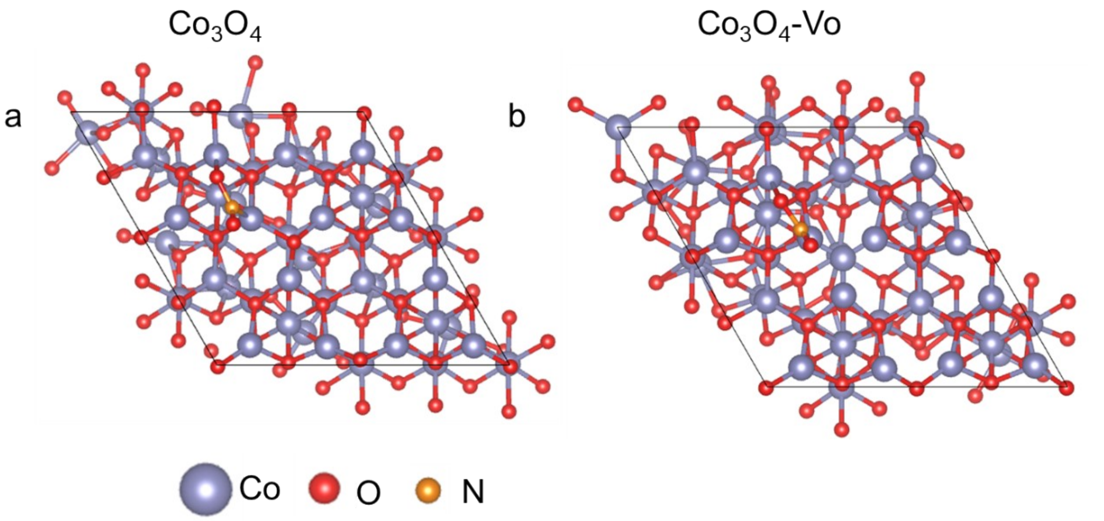

Figure S27. The adsorption structures of $^*NO_2$ on (a) Co_3O_4 (111) and (b) Co_3O_4-V_o (111) surface. The adsorption energy of NO_2^- over Co_3O_4 (111) and (b) Co_3O_4-V_o (111) surface was -2.88 eV and -3.39 eV, respectively.

Table S1 Comparison of FE, yield rate of urea and current density over CO_2 with different N source over Co_3O_4-1.0 with some state-of-the-art catalysts in electrochemical urea synthesis.

**Catalysts**	**N source**	**Potential (RHE)**	**J(mA/cm^2)**	**FE(%)**	**Yield rate mg·g^{-1}·h^{-1}**	**Ref.**
**Co_3O_4-1.0**	**NO_2^-**	**-0.7**	**34.1**	**26.35**	**3361**	**This work**
Te-doped Pd	NO_2^-	-1.1	8	12.2	358	4
L-Cu_1-CeO_2	NO_3^-	-1.6	64	5.29	3170	5
Cu-GS-800	NO_3^-	-0.9	27	28	1800	6
m-Cu_2O	NO_3^{--}	-1.3	20.9	9.43	1752	7
MoO_x/C	NO_3^-	-0.6	3.3	27.7	1432	8
Fe@C-Fe_3O_4/CNTs	NO_3^-	-0.65	3.7	16.5	1341	9
Cu-TiO_2-V_o	NO_3^-	-0.4	8	43.1	1248	10
B-FeNi-DASC	NO_3^-	-1.5	45	17.8	1212	11
V_o-CeO_2-750	NO_3^-	-1.6	47.2	5	943.6	12
PdCu/CBC	NO_3^-	-0.5	1.7	59.7	763.8	13
V_o-InOOH	NO_3^-	-0.5	1	51	592.5	14
$In(OH)_3$-S	NO_3^-	-0.6	1	53.4	533.1	15
AuPd	NO_3^-	-0.4			204.2	16
Co-PMDA	N_2	-0.5	1.5	48.97	868.2	17
Pd_1Cu_1-TiO_2	N_2	-0.5	1.9	22.54	600	18
$Ni_3(BO_3)_2$-150	N_2	-0.5	1.8	20.36	582	19
Bi-$BiVO_4$	N_2	-0.4	1.5	12.55	354.6	20
$BiFeO_3$/$BiVO_4$	N_2	-0.4	1.2	17.18	296.4	21
Pd_1Cu_1/TiO_2-400	N_2	-0.4	0.5	8.92	201	22
CuPc NTs	N_2	-0.6	1	12.99	143.7	23
MoP	N_2	-0.35	0.32	36.5	12.4	24
ZnO	NO	-0.92	40	11.26	907.8 ug/cm^2	25
ZnO-V_o	NO_2^-	-0.79	25	23.26	331.2 ug/cm^2	26
Cu@Zn	NO_3^-	-1.02	33.7	9.28	437.4 ug/cm^2	27
Ru-Cu	NO_3^-	0.13		25.4	151.6 ug/cm^2	28

References

1. G. Kresse and J. Hafner, *Journal of Physics: Condensed Matter*, 1994, **6**, 8245.
2. J. P. Perdew, K. Burke and M. Ernzerhof, *Physical review letters*, 1996, **77**, 3865.
3. S. Grimme, J. Antony, S. Ehrlich and H. Krieg, *The Journal of chemical physics*, 2010, **132**, 154104.
4. Y. Feng, H. Yang, Y. Zhang, X. Huang, L. Li, T. Cheng and Q. Shao, *Nano Letters*, 2020, **20**, 8282-8289.
5. X. Wei, Y. Liu, X. Zhu, S. Bo, L. Xiao, C. Chen, T. T. T. Nga, Y. He, M. Qiu, C. Xie, D. Wang, Q. Liu, F. Dong, C.-L. Dong, X.-Z. Fu and S. Wang, *Advanced Materials*, 2023, **35**, 2300020.
6. J. Leverett, T. Tran-Phu, J. A. Yuwono, P. Kumar, C. Kim, Q. Zhai, C. Han, J. Qu, J. Cairney, A. N. Simonov, R. K. Hocking, L. Dai, R. Daiyan and R. Amal, *Advanced Energy Materials*, 2022, **12**, 2201500.
7. M. Qiu, X. Zhu, S. Bo, K. Cheng, N. He, K. Gu, D. Song, C. Chen, X. Wei, D. Wang, Y. Liu, S. Li, X. Tu, Y. Li, Q. Liu, C. Li and S. Wang, *CCS Chemistry*, 2023, **0**, 1-11.
8. M. Sun, G. Wu, J. Jiang, Y. Yang, A. Du, L. Dai, X. Mao and Q. Qin, *Angewandte Chemie International Edition*, 2023, **62**, e202301957.
9. J. Geng, S. Ji, M. Jin, C. Zhang, M. Xu, G. Wang, C. Liang and H. Zhang, *Angewandte Chemie International Edition*, 2023, **62**, e202210958.
10. N. Cao, Y. Quan, A. Guan, C. Yang, Y. Ji, L. Zhang and G. Zheng, *Journal of Colloid and Interface Science*, 2020, **577**, 109-114.
11. X. Zhang, X. Zhu, S. Bo, C. Chen, M. Qiu, X. Wei, N. He, C. Xie, W. Chen, J. Zheng, P. Chen, S. P. Jiang, Y. Li, Q. Liu and S. Wang, *Nature Communications*, 2022, **13**, 5337.
12. X. Wei, X. Wen, Y. Liu, C. Chen, C. Xie, D. Wang, M. Qiu, N. He, P. Zhou, W. Chen, J. Cheng, H. Lin, J. Jia, X.-Z. Fu and S. Wang, *Journal of the American Chemical Society*, 2022, **144**, 11530-11535.
13. S. Zhang, J. Geng, Z. Zhao, M. Jin, W. Li, Y. Ye, K. Li, G. Wang, Y. Zhang, H. Yin, H. Zhang and H. Zhao, *EES Catalysis*, 2023, **1**, 45-53.
14. C. Lv, C. Lee, L. Zhong, H. Liu, J. Liu, L. Yang, C. Yan, W. Yu, H. H. Hng, Z. Qi, L. Song, S. Li, K. P. Loh, Q. Yan and G. Yu, *ACS Nano*, 2022, **16**, 8213-8222.
15. C. Lv, L. Zhong, H. Liu, Z. Fang, C. Yan, M. Chen, Y. Kong, C. Lee, D. Liu, S. Li, J. Liu, L. Song, G. Chen, Q. Yan and G. Yu, *Nature Sustainability*, 2021, **4**, 868-876.
16. H. Wang, Y. Jiang, S. Li, F. Gou, X. Liu, Y. Jiang, W. Luo, W. Shen, R. He and M. Li, *Applied Catalysis B: Environmental*, 2022, **318**, 121819.
17. M. Yuan, J. Chen, H. Zhang, Q. Li, L. Zhou, C. Yang, R. Liu, Z. Liu, S. Zhang and G. Zhang, *Energy & Environmental Science*, 2022, **15**, 2084-2095.
18. L. Pan, J. Wang, F. Lu, Q. Liu, Y. Gao, Y. Wang, J. Jiang, C. Sun, J. Wang and X. Wang, *Angewandte Chemie International Edition*, 2023, **62**, e202216835.
19. M. Yuan, J. Chen, Y. Xu, R. Liu, T. Zhao, J. Zhang, Z. Ren, Z. Liu, C. Streb, H. He, C. Yang, S. Zhang and G. Zhang, *Energy & Environmental Science*, 2021, **14**, 6605-6615.
20. M. Yuan, J. Chen, Y. Bai, Z. Liu, J. Zhang, T. Zhao, Q. Wang, S. Li, H. He and G. Zhang, *Angewandte Chemie International Edition*, 2021, **60**, 10910-10918.
21. M. Yuan, J. Chen, Y. Bai, Z. Liu, J. Zhang, T. Zhao, Q. Shi, S. Li, X. Wang and G. Zhang,

Chemical Science, 2021, **12**, 6048-6058.
22. C. Chen, X. Zhu, X. Wen, Y. Zhou, L. Zhou, H. Li, L. Tao, Q. Li, S. Du, T. Liu, D. Yan, C. Xie, Y. Zou, Y. Wang, R. Chen, J. Huo, Y. Li, J. Cheng, H. Su, X. Zhao, W. Cheng, Q. Liu, H. Lin, J. Luo, J. Chen, M. Dong, K. Cheng, C. Li and S. Wang, *Nature Chemistry*, 2020, **12**, 717-724.
23. J. Mukherjee, S. Paul, A. Adalder, S. Kapse, R. Thapa, S. Mandal, B. Ghorai, S. Sarkar and U. K. Ghorai, *Advanced Functional Materials*, 2022, **32**, 2200882.
24. D. Jiao, Y. Dong, X. Cui, Q. Cai, C. R. Cabrera, J. Zhao and Z. Chen, *Journal of Materials Chemistry A*, 2023, **11**, 232-240.
25. Y. Huang, R. Yang, C. Wang, N. Meng, Y. Shi, Y. Yu and B. Zhang, *ACS Energy Letters*, 2022, **7**, 284-291.
26. N. Meng, Y. Huang, Y. Liu, Y. Yu and B. Zhang, *Cell Reports Physical Science*, 2021, **2**, 100378.
27. N. Meng, X. Ma, C. Wang, Y. Wang, R. Yang, J. Shao, Y. Huang, Y. Xu, B. Zhang and Y. Yu, *ACS Nano*, 2022, **16**, 9095-9104.
28. J. Qin, N. Liu, L. Chen, K. Wu, Q. Zhao, B. Liu and Z. Ye, *ACS Sustainable Chemistry & Engineering*, 2022, **10**, 15869-15875.
